# Functional Architecture of the Inferior Colliculus Revealed with Voltage-Sensitive Dyes

**DOI:** 10.3389/fncir.2013.00041

**Published:** 2013-03-20

**Authors:** Lakshmi Chandrasekaran, Ying Xiao, Shobhana Sivaramakrishnan

**Affiliations:** ^1^Department of Anatomy and Neurobiology, Northeast Ohio Medical UniversityRootstown, OH, USA

**Keywords:** laminar organization, population coding, microcircuits, local circuits, post-inhibitory rebound

## Abstract

We used optical imaging with voltage-sensitive dyes to investigate the spatio-temporal dynamics of synaptically evoked activity in brain slices of the inferior colliculus (IC). Responses in transverse slices which preserve cross-frequency connections and in modified sagittal slices that preserve connections within frequency laminae were evoked by activating the lateral lemniscal tract. Comparing activity between small and large populations of cells revealed response areas in the central nucleus of the IC that were similar in magnitude but graded temporally. In transverse sections, these response areas are summed to generate a topographic response profile. Activity through the commissure to the contralateral IC required an excitation threshold that was reached when GABAergic inhibition was blocked. Within laminae, module interaction created temporal homeostasis. Diffuse activity evoked by a single lemniscal shock re-organized into distinct spatial and temporal compartments when stimulus trains were used, and generated a directional activity profile within the lamina. Using different stimulus patterns to activate subsets of microcircuits in the central nucleus of the IC, we found that localized responses evoked by low-frequency stimulus trains spread extensively when train frequency was increased, suggesting recruitment of silent microcircuits. Long stimulus trains activated a circuit specific to post-inhibitory rebound neurons. Rebound microcircuits were defined by a focal point of initiation that spread to an annular ring that oscillated between inhibition and excitation. We propose that much of the computing power of the IC is derived from local circuits, some of which are cell-type specific. These circuits organize activity within and across frequency laminae, and are critical in determining the stimulus-selectivity of auditory coding.

## Introduction

The inferior colliculus (IC) is the first major computational opportunity in the central auditory system, and provides information about the identity and location of a sound source. Extensive input convergence from most ascending and descending auditory areas (Oliver, 1984; Glendenning et al., 1992; Winer et al., 1998) gives the IC a critical position in auditory coding. In addition to external inputs, local circuitry within each colliculus (Oliver et al., 1991), commissural connections between colliculi (Aitkin and Phillips, 1984) and morphological and physiological cell-type heterogeneity (Oliver and Morest, 1984; Faye-Lund and Osen, 1985; Herrera et al., 1988; Paloff et al., 1992; Malmierca et al., 1993; Sivaramakrishnan and Oliver, 2001) generates a complex functional architecture that produces the broad range of responses to complex sounds that characterizes the IC (Irvine, 1992). In the same neuron, for instance, sounds can evoke excitatory and inhibitory synaptic potentials, and the timing and interactions of postsynaptic responses can be altered by varying the frequency or inter-aural properties of the sound (Nelson and Erulkar, 1963). The timing of synaptic and spike responses to free-field acoustic stimulation varies between neurons (Covey et al., 1996), and supra-threshold response properties are altered by inhibition (Kuwada et al., 1997). Understanding the basis of this extensive computing power is important for normal auditory processing and for pathological conditions where response constraints are compromised.

The anatomical architecture of the IC, with fibro-dendritic frequency laminae, cross-frequency projections, and bilateral tonotopic connectivity, provides a framework for complex acoustic processing. Laminae serve as modules of sound frequency, with rows of input axons making *en passant* synapses on neurons with aligned dendritic fields (Malmierca et al., 1993). Despite their narrow frequency range (Schreiner and Langner, 1997), however, laminae do not appear to be physiologically uniform, and exhibit systematic shifts in response characteristics such as onset latency (Langner et al., 1987) and threshold (Stiebler and Ehret, 1985). In addition to intra-laminar connections, inter-laminar connections within the same colliculus (Oliver et al., 1991) represent a major avenue for interactions between neurons tuned to different frequencies. Binaural processing also relies on commissural connectivity between the two colliculi, a major inhibitory source. Commissural fibers bilaterally connect laminae of the same best frequency (Saldaña and Merchan, 1992; Malmierca et al., 1995), and neurons activated by both lemniscal and commissural pathways (Moore et al., 1998) are distributed throughout the central nucleus (Adams, 1980; Aitkin and Phillips, 1984; Gonzalez-Hernandez et al., 1996). While the broad spectrum of functional architecture is well characterized, less is known about local interactions that create the functional fine structure and dynamical hierarchies that give the IC its computing power.

Determining functional connectivity patterns in the IC benefits from measuring responses simultaneously in distributed neuronal populations within and across laminae. While intrinsic optical signals (Higgins et al., 2010; Middleton et al., 2011) and calcium-sensitive dyes (Grienberger et al., 2012; Kubota et al., 2012) have been used to measure population activity in the auditory system, dyes that change their activity with membrane voltage measure the point of origin of responses and their propagation and have demonstrated the dimensions and functional organization of neural circuitry in the CNS (Blasdel and Salama, 1986; Horikawa et al., 1996; Huang et al., 2010). Voltage-sensitive dyes (VSDs) are membrane-bound molecules that change either their fluorescence or absorption (depending on the VSD used) on the same time scale as membrane voltage changes (<10 μs) (Loew et al., 1985), and responses are linear in the physiological range (Ross et al., 1977), allowing real-time measurements of activity. Here, we describe the use of VSD imaging to examine the functional architecture of the IC in brain slices, and demonstrate its viability in exploring activity in real-time within and across laminae. We further demonstrate that stimuli patterned to uniquely activate physiologically distinct cell types (Sivaramakrishnan and Oliver, 2001) reveal domains of microcircuits that are cell-type specific.

## Materials and Methods

### Preparation of laminar and transverse slices

**Figure 1:**
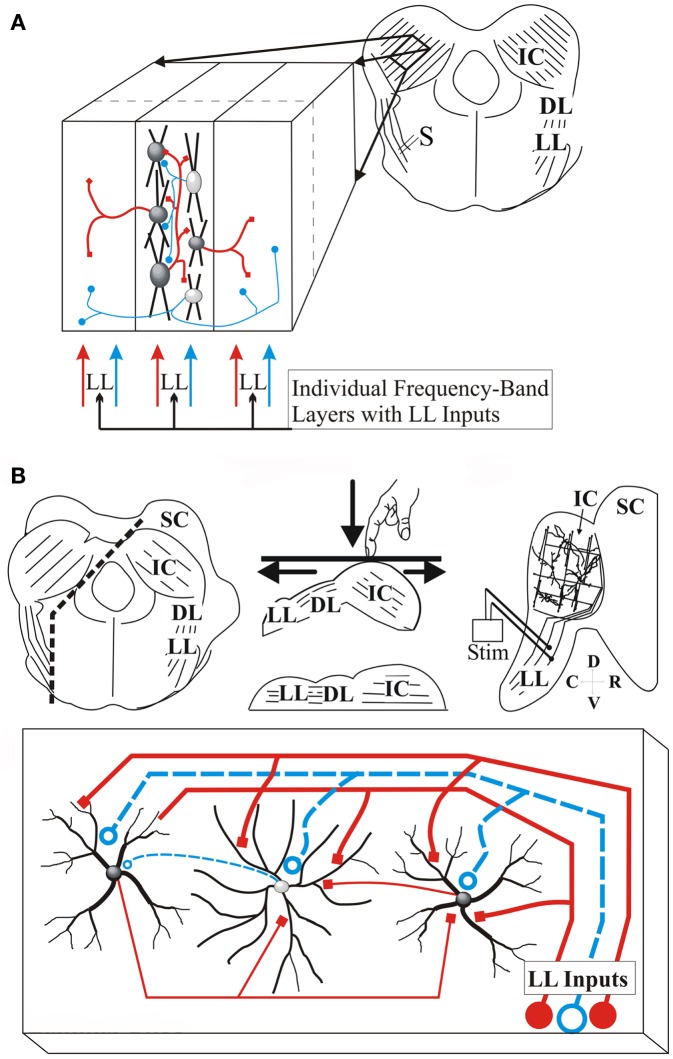
**Transverse and laminar slice planes of the IC**. **(A)** Schematic of the transverse IC slice. Right, slice plane with parallel arrangement of laminae; IC, inferior colliculus; LL, lateral lemniscus; DL, dorsal nucleus of the lateral lemniscus; S, Lemniscal stimulating electrode; Left, Expanded region of the slice illustrates laminar and cross-laminar arrangement with lemniscal input; Center section, arrangement of cells within a lamina with intra-laminar connections; Left and right sections, neighboring laminae receive inter-laminar connections; Red, excitatory; blue, inhibitory. **(B)** Schematic of laminar IC slice plane. Top, Transverse brain slab with additional cuts to generate a laminar slab; Left panel, Direction of the two cuts (dashed lines) made in the transverse brain slab; the lower cut parallel to LL afferents and the upper cut parallel to the laminae; middle panel, orientation of the brain slab on the surface of the vibratome cutting plate. The slab is pushed down so that the bottom surface straightens out, generating a single plane containing LL afferents and the IC. Right panel, the laminar slice; Only one side of the IC is present. SC, superior colliculus; Stim, LL stimulating electrode; Bottom, The laminar slice plane contains LL input fibers synapsing on rows of disk-shaped neurons, and local circuitry; Red, excitatory; blue, inhibitory (Reprinted and adapted with permission from Springer).

Long-Evans rats (P18–30) or CBA/Ca mice (P18–28) were used in experiments. Procedures were in accord with the regulations of the Institutional Animal Care and Use Committee at the Northeast Ohio Medical University and guidelines of the National Institutes of Health. Techniques for making transverse and laminar brain slices through the IC have been previously described (Sivaramakrishnan et al., 2004; Sivaramakrishnan and Oliver, 2006) and will be briefly outlined here. To obtain brain slices, animals were anesthetized with isoflurane and then decapitated. A block containing the IC was removed from the brain with two transverse cuts. For transverse slices, the block was glued to the cutting stage of a vibratome (Dosaka, Japan) with the superior colliculus facing down and slices made parallel to the surface of the brain block (Figure 1A). For laminar slices, two additional cuts were made in the brain block, one parallel to the lateral lemniscus in the parasagittal plane and a second at 45° to the sagittal plane, parallel to the ICC laminae. The cut surface was aligned on a vibratome stage with the lemniscus and IC in the same plane (Figure 1B). With this orientation, the first 80–100 μm of tissue, consisting mainly of the external cortex of the IC, was discarded. Slices were 150 μm thick in both the transverse and laminar planes. Slices were made in oxygenated (95% O_2_/5% CO_2_) artificial cerebrospinal fluid (ACSF) (in mM: 120 NaCl, 3 KCl, 2 CaCl_2_, 1.3 MgSO_4_, 1 NaH_2_PO_4_, 20 NaHCO_3_, 25 glucose, pH 7.4) and cut, stored and recorded from at 35°C. For recordings, slices were transferred to a temperature-regulated recording chamber and superfused with ACSF at 2 ml/min. Antagonists of the AMPA receptor (NBQX; 10 μM), the GABA_A_ receptor (SR95531; 20 μM), and tetrodotoxin (TTX; 1 μM), a blocker of voltage-gated sodium channels, were bath applied at the same flow rates as ACSF. Chemicals were obtained from Sigma-Aldrich. Data are reported from 102 slices (72 rat IC, 30 mouse IC) from 33 rats and 21 mice.

### Electrical stimulation of the lateral lemniscal tract

The lateral lemniscal (LL) nerve bundle was stimulated with an extracellular concentric bipolar electrode made from tungsten/platinum wire, with an active diameter of 100 μm. In both the transverse and laminar slices, the stimulus electrode was placed on the LL tract before it entered the IC (Figure 1). The location of the LL stimulus electrode allowed stimulation of LL fibers of passage from lower brainstem nuclei as well as those from the dorsal nucleus of the LL. Stimulation was performed with a multi-channel stimulator (AMPI) through a constant current isolation unit (WPI A365). The biphasic stimulus currents used created negligible net DC current flow between pulses and prevented build-up of current on the LL tract during stimulus trains. Stimulus parameters were modified as needed to evoke specific response patterns. For “minimal” stimulus paradigms, stimulus currents and durations were kept low (<0.3 mA; 0.1–0.3 ms); in other experiments, stimulus currents or durations were increased as necessary.

### Measurement of voltage-sensitive dye signals in IC brain slices

Slices were incorporated with the oxonol VSD NK3630 (first synthesized by R. Hildesheim and A. Grinvald as RH482; available from Nippon Kankoh-Shikiso Kenkyusho Co., Ltd., Japan; See Momose-Sato et al., 1999 for molecular structure) dissolved in ACSF to a final concentration of 5–10 μg/ml. A staining period of 90–120 min produced even staining in the slice. After staining, slices were washed in dye-free ACSF for at least 30 min. before experiments. The slice was trans-illuminated by light from a 100 W tungsten-halogen bulb passing through a 705 nm (BW 15 nm) filter to measure peak absorbance or a 640 nm (BW 15 nm) filter (Chroma) as a control. The slice was exposed to the 705 nm wavelength only during recording, for a maximum of 2 s/trial and <50 trials per slice. With this level of exposure, photodynamic damage and dye bleaching were minimal. Images were collected with a 464-element photodiode array of optical detectors and eight additional analog channels (WuTech instruments; www.wutech.com). Frame rates with this array are ∼1600 frames/s (inter-frame interval of ∼0.614 ms). The diode array was mounted on an Olympus microscope fitted with 5× (dry, NA 0.1) and 20× (water immersion, NA 0.95) objective lenses. The optical signal from each detector was individually amplified 200 times, low-pass filtered at 333 Hz, and then multiplexed and digitized with 12 bit at 1600 samples/s/channel. Data were collected and analyzed using NeuroPlex software (RedShirtImaging, Fairfield, CT, USA) and Origin (OriginLab) and displayed as traces for numerical analysis and pseudocolor images for still time-lapse and real-time activity patterns. A digital camera fitted on to a second port was used to photograph the slice, on which VSD images were superimposed. Field potentials, measured with a tungsten electrode, were passed through a differential amplifier (A-M Systems) and fed into an auxiliary (BNC) channel of the data-acquisition interface board. In the recordings reported in this study, the field electrode was used only as an indicator of the stimulus current and did not provide any direct information about cellular activity. To minimize visual obstruction within the ICC, the field electrode was placed in the dorsal or external cortices in transverse slices and at the rostral or caudal edges of laminar slices.

**Figure 2:**
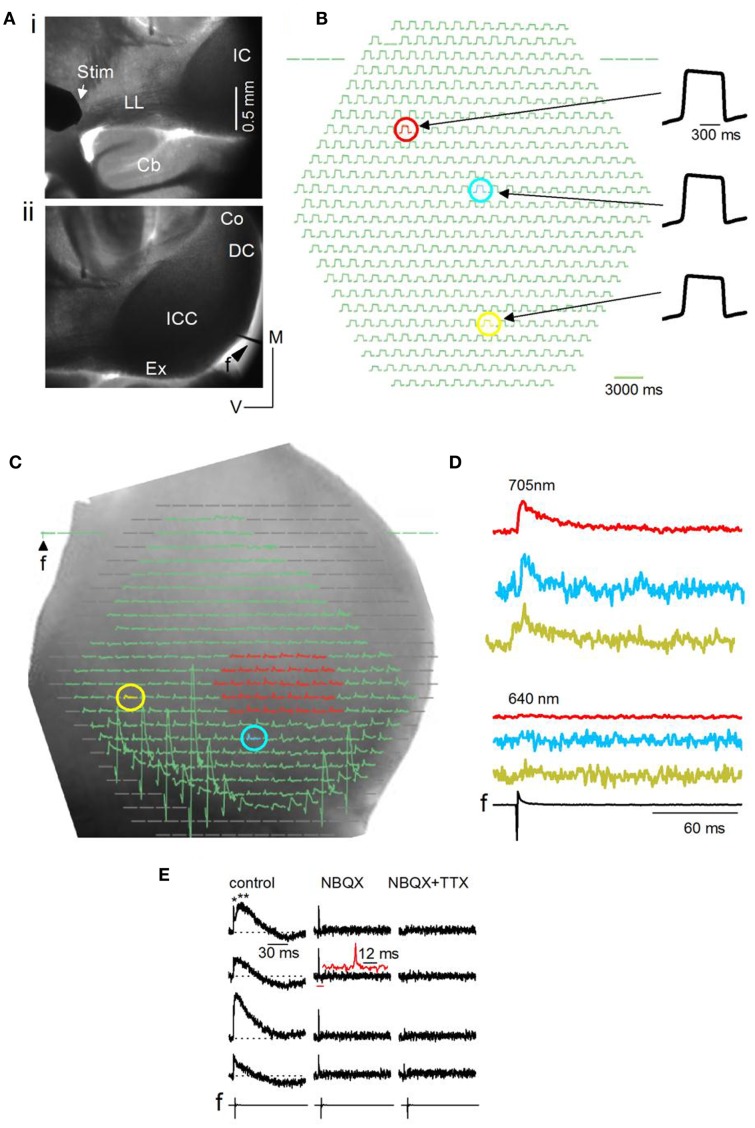
**General methods and artifactual considerations for the use of VSDs in IC brain slices**. **(A)** Image plane of one side of the inferior colliculus (IC) (mouse, P23) viewed through a 5× objective. Transverse slice, 150 μm thick. (i) Furthest possible placement of the stimulating electrode (Stim) on the lateral lemniscal (LL) tract. Cb, cerebellum. (ii) Field electrode (*f*) at the dorsal edge of the slice. ICC, central nucleus; Ex, external cortex; DC, dorsal cortex; Co, commissure. **(B)** Resting light intensity (RLI) for a small area of the ICC; 20× objective. The RLI is the light absorbed by the VSD in the un-stimulated state. The red, blue, and yellow pixels show slight differences in maximum signal amplitude. **(C)** Diode array coverage of one side of the IC (rat) in a transverse slice. 5× objective. *f*, field electrode input. Blanked pixels outside the edge of the slice or the region of interest are omitted before analysis. Regions of interest for analysis are color-coded. Red, a group of multiple pixels over which responses are averaged; blue and yellow, different single pixels. The RLI has been subtracted before displaying this image. **(D)** VSD responses evoked by a single LL shock displayed as traces. Traces correspond to the red area and individual yellow and blue pixels selected in **(C)**. Absorbance measured through a 705 nm filter (top) and a 640 nm filter (bottom). *f*, field recording. **(E)** VSD signals evoked by a single LL shock are shown at different detectors covering the IC central nucleus. Left panel, normal ACSF; middle panel, 10 μM NBQX (an antagonist of glutamatergic AMPA receptors); right panel, 10 μM NBQX + 1 μM TTX. LL shock strength 3 mA. Left panel, top trace, single and double asterisks point to the first fast and second slower components; Inset, second trace from top, expanded region corresponds to the horizontal red bar; The 12 ms scale bar is associated with the inset.

Responses in the IC were evoked by activating afferent input through stimulation of the LL nerve tract with an extracellular bipolar electrode placed on the tract before it entered the IC (Figure 2Ai). With a 5× objective, one side of the IC is visible, extending sometimes to the beginning of the commissural region depending on the age of the animal and the species (mouse or rat) (Figure 2Aii). The field electrode, *f*, was placed in the dorsal or external cortices. NK3630 preferentially incorporates into neuronal membranes, with little glial contamination (Konnerth et al., 1987). We confirmed glial insensitivity in the IC by recording responses before and after blocking glial transporter currents with dl-threo-β-aspartic acid (Kojima et al., 1999) and detected no measureable change in the absorbance signal [*n* = 6 slices; average over different regions of the IC central nucleus; *t*(5) = 1.31; *p* = 0.23].

To assess the uniformity of dye penetration in the slice and to control for changes in signal magnitude with bleaching, the relative light intensity (RLI; the background absorbance in the slice) was measured before an experiment and between recordings. The RLI is generally 0.01–0.001 of the illumination intensity (the light intensity that the tissue receives), and is about 10^7^ photons/ms/detector (Lippert et al., 2007). A fairly uniform RLI suggested adequate dye penetration through the slice (Figure 2B, compare response magnitudes of red, blue, and yellow pixels). The RLI was either subtracted from the VSD signal or the VSD signal measured as a ratio (VSD/RLI); we did not notice significant differences in the net change in the stimulus-evoked signal between these methods.

Before analysis, pixels at the edge of the diode array, those covering regions beyond the IC, and pixels covered by the field electrode (Figure 2C) are blanked, limiting analysis of the VSD signal to the IC or smaller regions within it. Activity may be summed or averaged over several pixels (Figure 2C, e.g., red pixels) or measured in single pixels (Figure 2C, e.g., blue and yellow pixels) and plotted as traces (Figure 2D). We perform several controls to detect artifactual changes in the VSD signal. Uneven fluid movement over the slice during bath perfusion, which produces varying path lengths for light transmitted to the objective, is minimized by turning off perfusion for 30 s before LL stimulation and, in addition, switching absorbance filters between the 705 nm (BW15 nm) filter used with NK3630 (Figure 2D, top traces) to a 640 nm (BW15 nm) filter, which filters out the NK3630 signal (Figure 2D, bottom traces). A second potential source of artifact is phototoxicity. NK3630 has been reported to have almost no toxicity at the concentrations we used (Xu et al., 2010), however, we tested for toxicity by comparing the field potential and VSD signals before and after turning on the transmitted light and placing the 705 nm filter in the light path. Repeated trials showed no change in either recording, confirming a lack of toxicity in IC slices (*n* = 4 slices; *p* = 0.2). A third source of artifact, which arises from direct transmission of the electrical current from the LL stimulus electrode, is minimized by placing the stimulus electrode well away from the IC (as in Figure 2Ai) and using stimulus currents <5 mA. VSD signals in the central nucleus of the IC often consist of an initially rapid response followed by a slower one (Figure 2E, left column, single and double asterisks). To control for stimulus current-related artifacts that may give rise to the rapid signal, in addition to switching filters between 705 and 640 nm, we apply the glutamate AMPA receptor blocker NBQX followed by the sodium channel blocker TTX. The slow response is abolished by NBQX, leaving a rapid response with the shape of a spike (Figure 2E, middle column; inset, second trace from top), that is abolished by the further addition of TTX (Figure 2E, right column), suggesting a sodium spike. Thus, provided camera frame rates are fast enough, the ability of NK3630 to distinguish between spikes and synaptic potentials in IC slices, even at low magnifications (5×), makes it a viable means of measuring fine temporal structure within activity patterns.

### Data-acquisition and analysis

The strength (current magnitude × duration) and frequency of LL stimulation were critically varying parameters in these experiments. For minimal stimulation experiments, stimulus strength was carefully adjusted, first, to obtain responses throughout the central nucleus of the IC and second, to prevent spikes in single pixels. This adjustment was made for every slice. The optimal current strength was a 0.1–0.3 ms, 0.1–0.3 mA pulse(s). Data were analyzed only if the single-pixel signal to noise ratio, averaged over the entire ICC within the field of view, was >3. For other experiments, stimulus strengths were altered as needed. Stimulus train frequencies were also adjusted, again in each slice, to generate the responses needed. For example, rebound circuits described in Figure 14 were isolated by stimulating the LL with 20–40 Hz trains >400 ms long, using shock strengths <0.2 mA. This stimulus pattern allowed for the gradual build-up of a net hyperpolarizing response that was required to generate rebound spiking. Stimulus parameters were adjusted for each slice by examining the responses in all the pixels covering the ICC; stimulus currents were within a 0.1 mA range across different slices.

In minimal stimulation experiments, responses summed over multiple pixels were compared to responses in single pixels. Multiple pixels in each area were clustered, and the number of pixels and the region of slice they covered were kept constant between slices. We used several slices to obtain an optimum estimate of the number of pixels that would adequately represent high, middle, and low frequency regions within slices. For transverse slices, we found that 16 pixels were optimum within one slice as well as for the sample of slices used for data analysis. The selection of this number of pixels and the area they covered were determined using four main criteria: (1) the extent of coverage of each frequency region in the slice, (2) the consistency of the number of pixels covered by each frequency region in different slices, (3) no overlap between frequency regions, and (4) no loss of pixels at the edge of the slice. In the 26 transverse slices from which data are reported in Figures 4–6, data were analyzed using the same 4 × 4 arrangement of the 16 pixels within each topographic region in every slice. In 5 of the 26 transverse slices, one pixel in the ventral ICC (in each of three slices), and two pixels in the dorsal ICC (one in each slice) did not take up the dye and therefore did not contribute to the data. In these slices, data was analyzed from 15 pixels in the regions missing the single pixel and 16 pixels in the regions with no loss of dye. The loss of data from one pixel did not affect results significantly. In laminar slices, we used 38 pixels to define each region in both the ventral-dorsal and rostral-caudal directions. The selection of this number of pixels allowed adjacent regions of interest to be separated by two rows of pixels. To control for deterministic bias, we performed multi-pixel analysis by choosing other arrangements of clustered pixels within each region of interest, one example of which is demonstrated in Figure 4B. These could include as many as 40 pixels or as few as six pixels within each cluster. As long as the number of pixels was kept the same between regions, the results did not vary from the 16 pixel (transverse) or 38 pixel (laminar) clusters used in this study. Further, although we used clusters of pixels, results did not differ if random pixels were selected in an area, as long as the number of pixels between slice regions was the same. The equivalence of data obtained from different multi-pixel configurations was only possible with minimal stimulation experiments designed to evoke sub-threshold responses in individual pixels. Single-pixel analysis was performed on individual pixels, and as an average of the 16 or 38 single pixels within the same cluster used for multi-pixel analysis. For both multi- and single pixel analyses, responses within a slice were first averaged from multiple trials, and then further averaged over the sample of transverse or laminar slices.

The placement of the field electrode, which was used to record the stimulus artifact, and the position of the stimulating electrode on the LL tract varied between slices, thus all multi-pixel latencies were measured with respect to the average value of the multi-pixel onset latency in the ventral ICC. This average was determined over the same set of multiple pixels used for multi-pixel analyses. Latencies of onsets of the different peaks in different topographic regions were measured from the multi-pixel onset latency within that region. Because the amplitude of the VSD signal has arbitrary units, peak amplitudes were compared by combining multi- and single-pixel responses into one data set, and expressing all peak heights as a ratio of the peak height of the multi-pixel response in the ventral ICC. Response durations were compared using absolute values measured at half the maximum response amplitude.

Results are expressed as mean ± SEM. SD, when used, is indicated in the text. Significance was determined using Student’s *t*-test or ANOVA where pertinent; *p* < 0.05 with the Bonferroni correction factor applied. *p* and *F*(*df*1, *df*2) values are indicated in the text. Trials were repeated several times (4–10 trials) on a single slice, and the average of that data taken as the data for that particular slice. This was done for each slice in our sample. The averaged data from each slice was further averaged across all the slices. Means and SEs were calculated, significance determined, and ANOVA was performed on data both within a slice and on the pooled data from the sample.

## Results

We describe the use of VSD imaging in transverse and laminar slice planes to examine spatio-temporal patterns of lemniscally evoked activity across and within frequency regions.

### Activity in the inter-laminar plane of the IC

Transverse planes of the IC preserve inter-laminar connections and minimize connections within a lamina, which are limited mainly to the dorso-ventral extent of that lamina within the slice (Oliver et al., 1991). Commissural fibers are intact in this slice plane, connecting laminae of the same best frequency in the two colliculi (Saldaña and Merchan, 1992; Malmierca et al., 1995). Recordings in the gerbil IC show that most neurons are activated by both lemniscal and commissural pathways (Moore et al., 1998). Commissurally derived inhibition is primarily GABAergic, whereas lemniscal afferents provide both GABA and glycinergic inputs (Moore et al., 1998) with monosynaptic and polysynaptic components (Wagner, 1996; Moore et al., 1998). The transverse slice plane has been used extensively for recordings from single neurons in the central nucleus of the IC (ICC) in response to direct current injection, and lemniscal and commissural synaptic input (Moore et al., 1998; Peruzzi et al., 2000; Sivaramakrishnan and Oliver, 2001; Sivaramakrishnan et al., 2004). Incoming LL axons can be stimulated in this slice plane, with placement of the stimulus electrode allowing activation of afferent input from the dorsal nucleus of the LL and from lower brainstem centers (Figure 1A).

#### Synaptically evoked activity within the ipsilateral colliculus

**Figure 3:**
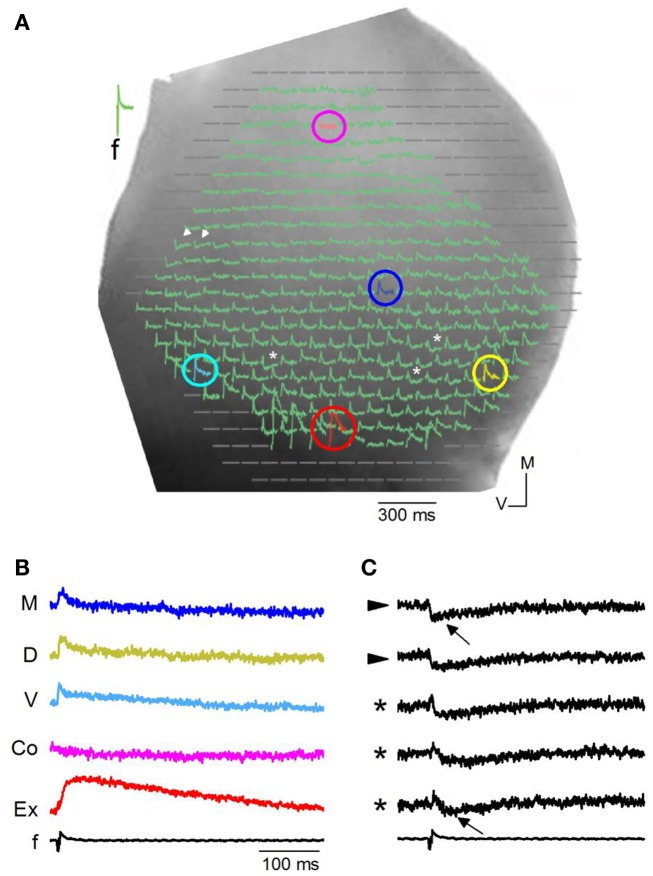
**Responses in a transverse slice evoked by minimal LL stimulation**. **(A)** Image of one side of a rat IC seen through a 5× objective with diode array superimposed. Colored circles, pixels with depolarizing responses. Pink, commissure; dark blue, medial ICC; yellow, dorsal ICC; red, external cortex; light blue, ventral ICC. White arrowheads, pixels with hyperpolarizing responses; asterisks, pixels with mixed de- and hyperpolarizing responses. **(B)** Traces of depolarizing VSD responses corresponding to the color-coded pixels in **(A)**. M, medial ICC; D, dorsal ICC; V, ventral ICC; Co, just lateral to the commissural region; Ex, External cortex; *f*, field recording. **(C)** Traces of hyperpolarizing and mixed de- and hyperpolarizing VSD responses corresponding respectively to the arrowheads and asterisks in **(A)**. Arrows, peak hyperpolarization.

**Figure 4:**
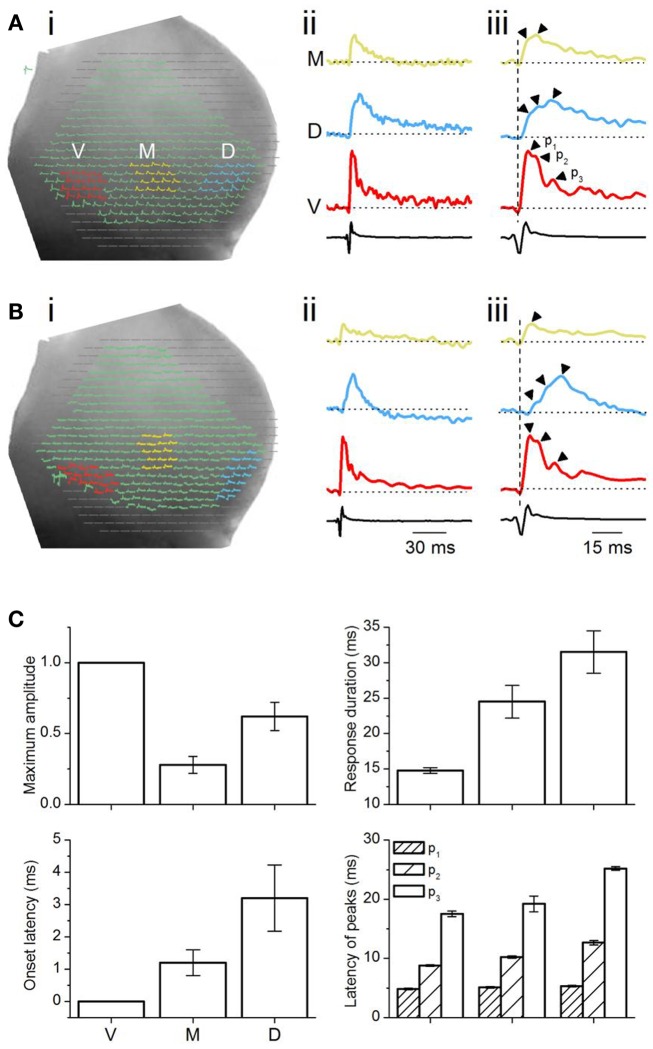
**Analysis of multi-pixel responses in the transverse plane**. **(A,B)** (i) Two slices illustrating different sets of 16 pixels chosen in the ventral (red), medial (yellow), and dorsal (light blue) regions of the ICC. (ii) Responses summed over the 16 pixels in each area. M, medial ICC; D, dorsal ICC; V, ventral ICC. (iii) Expanded illustrations of the traces in (ii). Arrowheads by each trace: peaks in the response. *p*_1_, *p*_2_, *p*_3_, peak 1, peak 2, peak 3; Dotted vertical line, time of response onset in the ventral ICC. Scale bars apply to traces in **(A,B)**. **(C)** Multi-pixel measures of maximum amplitudes, duration of response, onset latency, and times of subsequent peaks. Maximum amplitudes were measured as a fraction of the maximum amplitude in the ventral ICC. Response durations are absolute values, measured at half-maximum amplitude. Onset latencies were measured with respect to the onset of the response in the ventral ICC, which was set to zero. The latencies of peaks were measured with respect to the onset latency in each region. Each point is the average of three to four trials from each slice, further averaged over 26 slices, with the exception of *p*_3_ in the medial ICC (average of 14 slices). Error bars are SEM.

**Figure 5:**
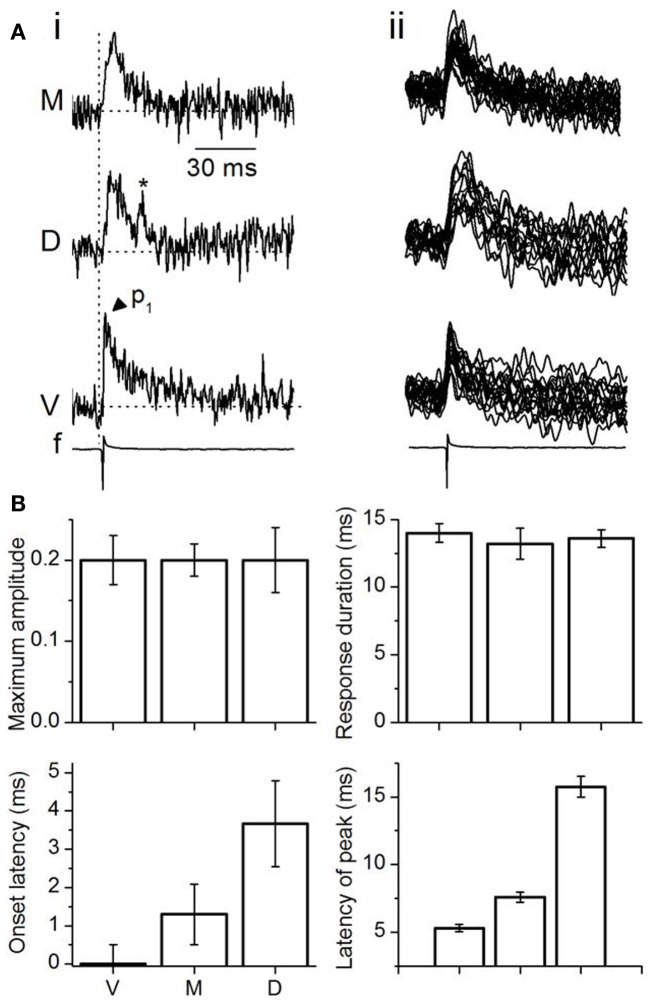
**Analysis of single-pixel responses in the transverse plane**. **(A**) (i) LL-evoked responses in single pixels from different ICC regions. M, medial; D, dorsal; V, ventral ICC; *f*, field recording. Vertical dotted line, time of response onset in the ventral ICC; *p*_1_, single-peak; Asterisk, spontaneous event. (ii) Superimposed traces from 16 single pixels in each region illustrated for the same slice as (i). The 16 pixels picked are the same ones used for multi-pixel analysis. **(B)** Single-pixel measures of maximum amplitudes, duration of response, onset latency, and time of single-peak. Maximum amplitudes are depicted as fractions of the maximum amplitude of the multi-pixel response in the ventral ICC. Response durations were absolute values, measured at half-maximum amplitude. Onset latencies were measured with respect to the onset of the multi-pixel peak response in the ventral ICC, which was set to zero. Peak time of responses in individual pixels was measured with respect to the average single-pixel onset latency in each region. Averages are from the 16 pixels used for multi-pixel analysis in each region of the slice. Each point is the average of three to four trials from each slice, further averaged over 26 slices. Error bars are SEM.

**Figure 6:**
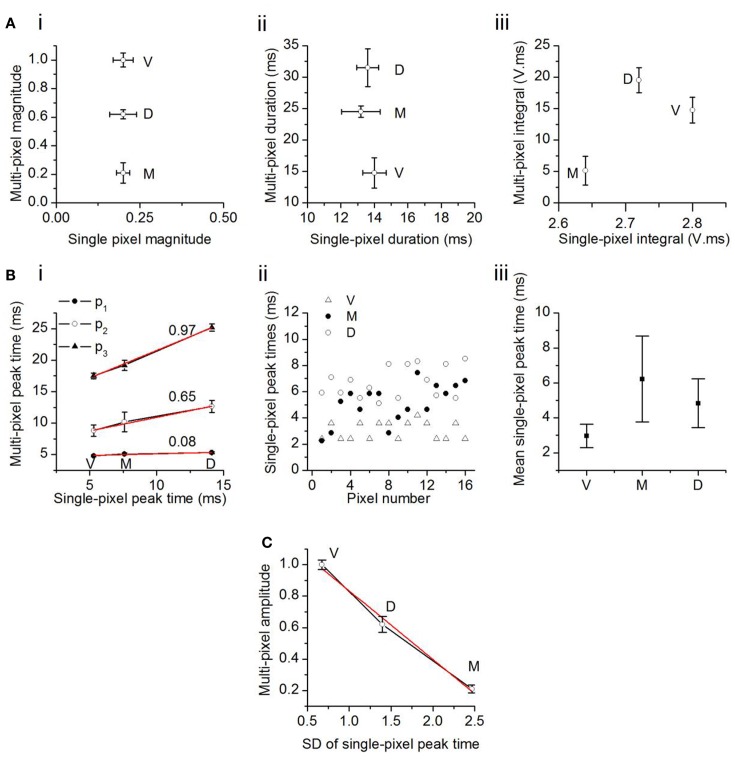
**Comparison between multi- and single-pixel responses in the transverse plane**. **(A)** Multi-pixel measures as functions of the same measures in single-pixel responses. (i) Response magnitude; (ii) duration; (iii) integral of magnitude and duration. The component V in the unit of integral, V. ms, is a fraction of the maximum response in the ventral ICC, and is unitless. Twenty-two slices; mean and SEM. **(B)** (i) Times of onset of the first, second, and third multi-pixel peaks as a function of the single pixel peak time. Linear fits (*r*^2^: *p*_3_, 0.998; *p*_2_, 0.985; *p*_1_, 0.949) with slopes as indicated. V, ventral; M, medial; D, dorsal ICC. (ii) Distribution of single pixel peak times in the ventral, medial, and dorsal regions in one slice. Pixels chosen are the same 16 pixels used for multi-pixel analysis in that slice. (iii) Mean single-pixel peak time in the ventral, medial, and dorsal ICC. Values are mean and SD of data from 14 transverse slices. **(C)** Maximum amplitude of the multi-pixel response plotted as a function of the SD of the scatter in single pixel peak times. Data from 14 transverse slices. Error bars are SEM. Red line, linear fit, *r*^2^ = 0.987.

**Figure 7:**
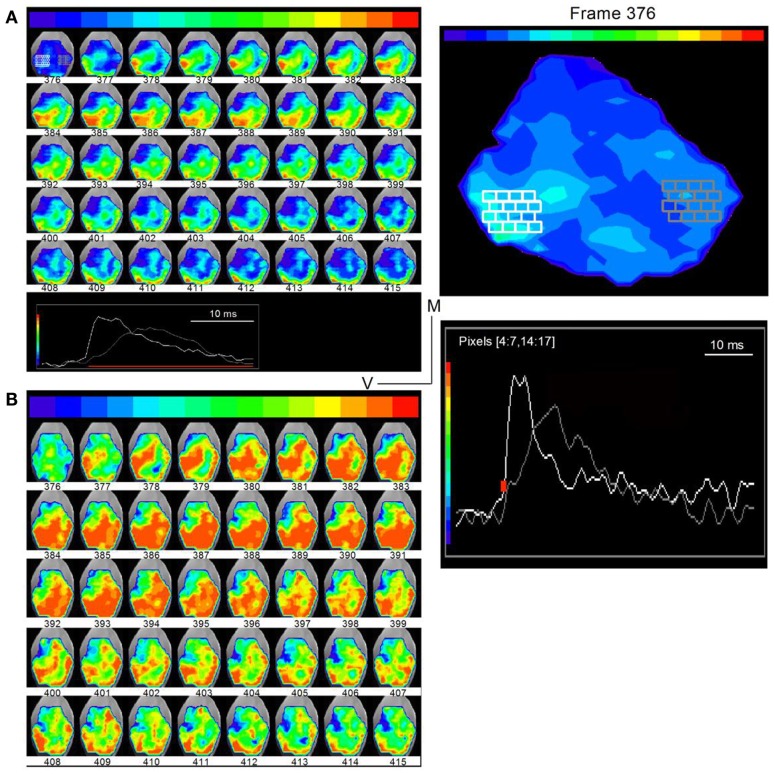
**Direction of lemniscal and cross-laminar propagation**. Time lapse images of responses in a transverse slice superimposed on a photograph of the IC (rat, P21) taken through a digital camera on a second port of the microscope. Frame numbers are indicated below each frame. Frames are 0.6 ms apart. **(A)** Low-gain images show primarily the direction of lemniscal propagation. Inset at bottom: white and gray traces are the summed responses in the corresponding white and gray pixelated areas in the first image (frame 376). Right panel, Expanded image of the first frame, 376, with inset traces of averaged responses in white and gray pixelated areas. The areas picked are used to illustrate different regions of the slice. The region displayed between frames 376 and 415 occur during the region of the response depicted by the red line in the inset. **(B)** High-gain images of the same time-lapse series show activity in directions that would correspond to cross-frequency connections. The frame sequence is the same as in **(A)**.

**Figure 8:**
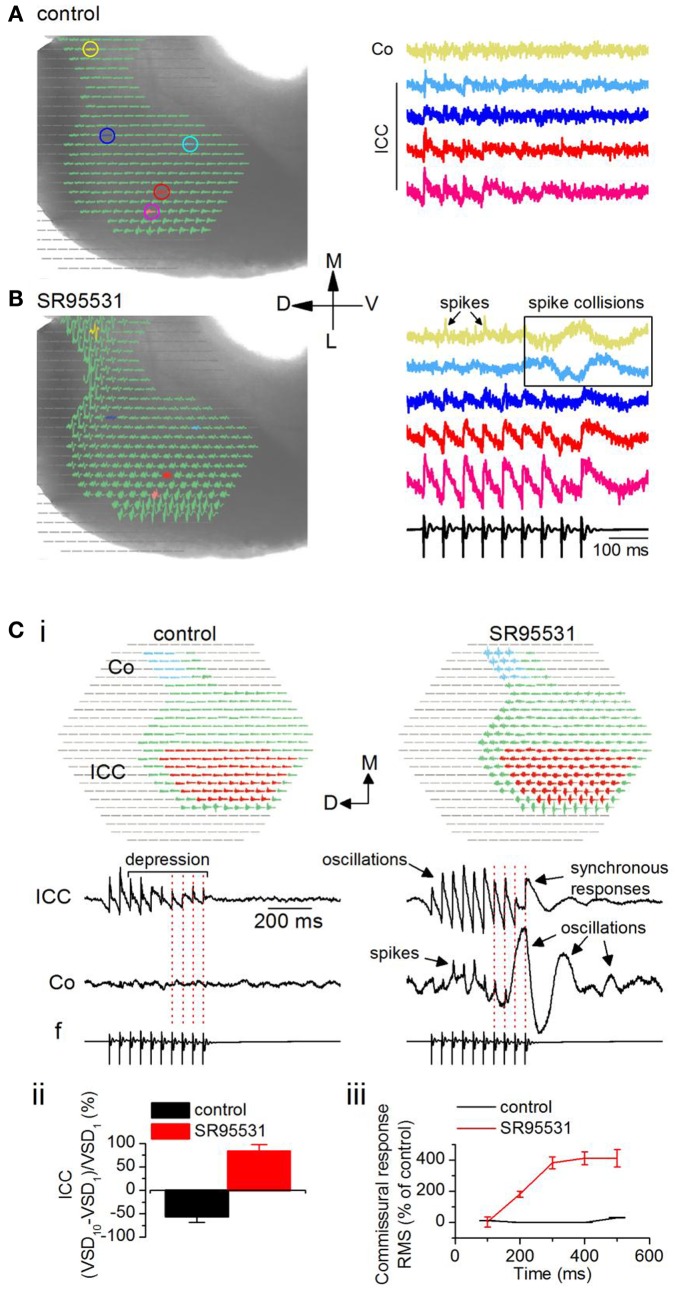
**Loss of inhibition promotes inter-collicular connectivity**. Responses in a transverse IC slice (mouse, P18). **(A)** Normal ACSF. **(B)** SR95531 (an antagonist of GABA_A_ receptors; 20 μM). **(A,B)** Left panels, Diodes chosen to be displayed include the commissural region (yellow) through the lateral region of the ICC. Pixels at the lateral edge of the slice in the region of the external cortex have been omitted from the display. Right panels, Traces corresponding to the colored pixels in left panels. Traces are averages of eight trials in control and six in SR95531. Boxed region highlights non-linear activity profiles in the commissure. Co, commissure; ICC, IC central nucleus. **(C)** Effect of SR95531 on time-dependent changes in commissural activity during a 20 Hz stimulus train. (i) Left panel, control; right panel, SR95531. Top, regions in the ICC and the commissural area (Co) over which responses are averaged. Bottom, Averaged responses illustrated as traces. *f*, field recording. Vertical dotted lines are aligned to the shocks in the field recording. (ii) Depression/facilitation index for responses in the ICC in control and SR95531. VSD_1_, VSD_10_, responses to the first and 10th shocks in the stimulus train. (iii) Root mean square (RMS) of the response integral in the commissural region plotted as a function of sequential 100 ms time windows during the response. The 100 ms data point is the integral of the response between 0 and 100 ms; the 200 ms data point is the integral of the response between 100 and 200 ms, and so on. Data in (ii) and (iii), 19 slices, mean and SEM.

**Figure 9:**
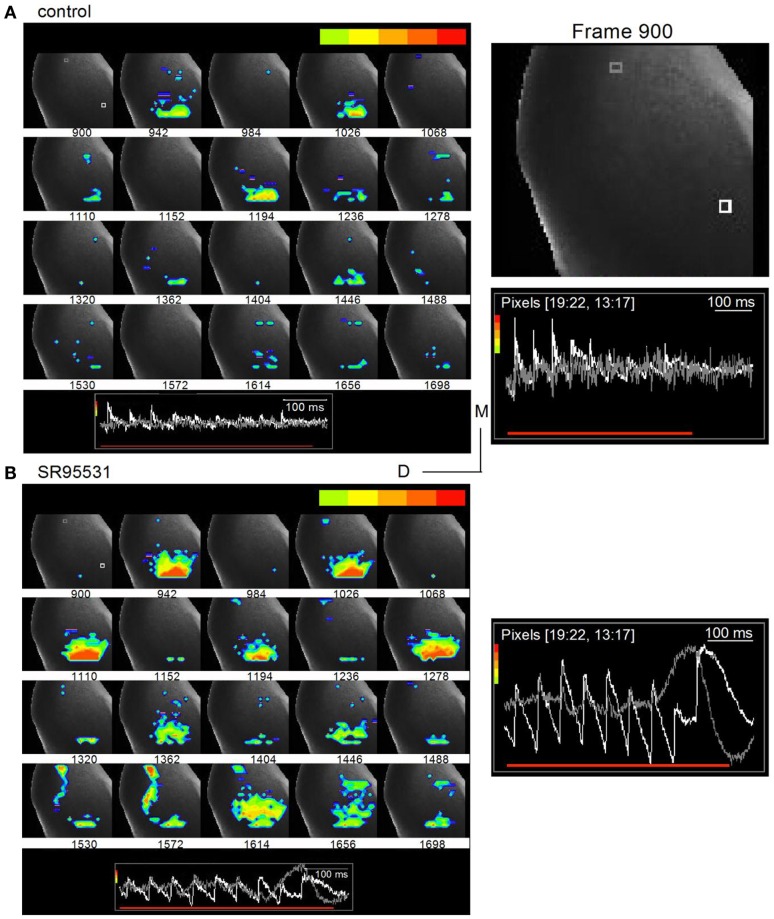
**Commissural propagation in the presence of GABA_A_ antagonists**. Time lapse images of the response during a 20 Hz train superimposed on an image of the IC. The color bar at the top of each panel shows the range of responses illustrated is restricted to the peak response regions to isolate commissural propagation from the more diffuse spread of activity in the ICC. **(A)** Normal ACSF. Right panel, Expanded image of the first frame, 900, with inset traces of responses in single white and gray pixels. White pixel, ICC; gray pixel, commissure. Frames are 25 ms apart. **(B)** 20 μm SR95531. Insets, traces correspond to the white and gray pixels highlighted in the first frame (frame 900) in the top panel. The gray pixel is in the commissural region. Right panel, Expanded image of inset traces of responses in the white and gray pixels. Pixelated areas in frame 900 [**(A)**, right panel] also applies to **(B)**. Frame intervals are the same as in **(A)**. Directional scale bar applies to both images.

**Figure 10:**
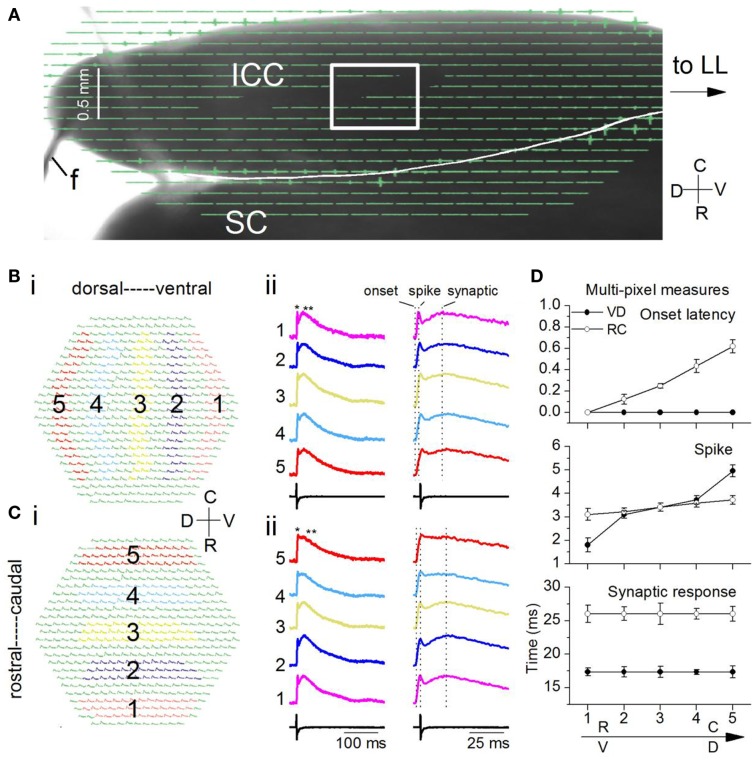
**Spatio-temporal activity patterns within a frequency lamina**. **(A)** Image of the laminar slice (mouse IC; P19) seen through the diode array with a 5× objective. The LL is out of the field of view. *f*, field electrode. Light gray line across the dorsal edge of the slice is a string from the slice anchor. Box, region of the ICC analyzed with a 20× objective. SC, superior colliculus. **(B,C)** Responses to a single LL shock. Average of 10 trials. **(B)** (i) Areas chosen in the ventral (1) and moving incrementally toward the dorsal (5) region of the ICC. The same number of pixels are chosen in each area. (ii) Left columns, Traces corresponding to the color-coded areas in the ventral-dorsal and rostral-caudal directions; single asterisk, spike; double asterisk, synaptic response; Right columns, expanded traces to show onset latencies and peak times (vertical dotted lines). **(C)** (i) Areas in the rostral (1) toward the caudal (5) region. (ii) Left and right columns are as in **(B)**. **(D)** Response times along the ventral-dorsal (V-D) and rostral-caudal (R-C) directions. Top, onset; middle, first peak/spike; bottom, second peak/synaptic response. Times were measured with respect to onset in the ventral or rostral ICC (area 1). Each point is the average of 3–10 trials in each of 14 slices. Error bars are SEM.

**Figure 11:**
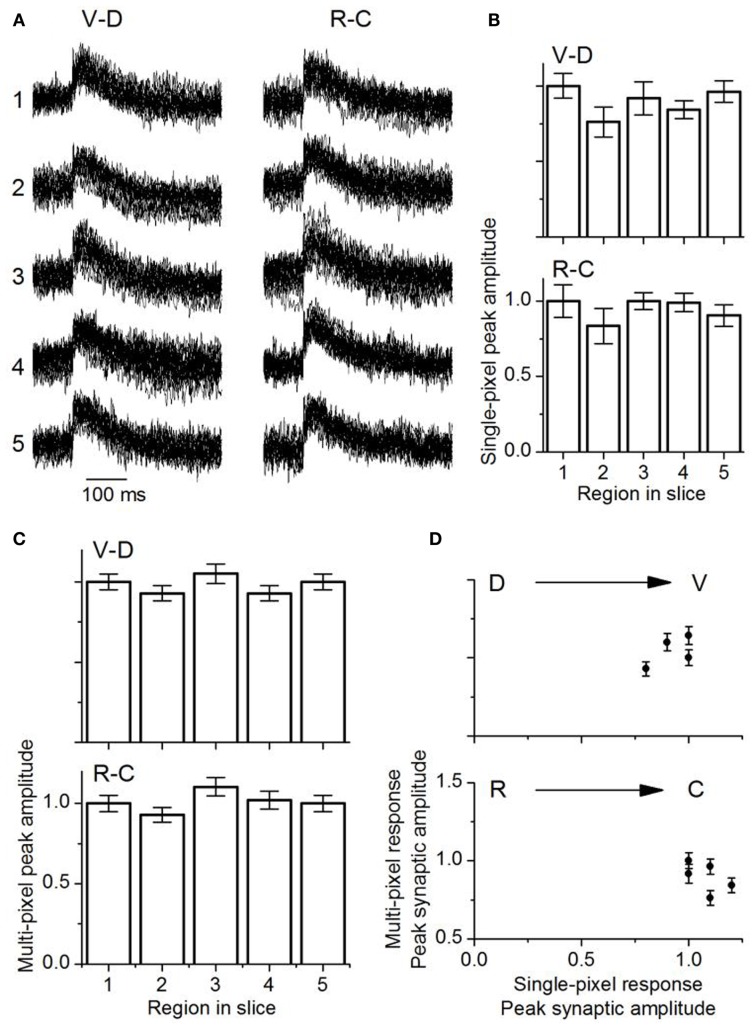
**Comparison of multi-pixel and single-pixel responses in the laminar plane**. **(A)** Overlapped traces from 16 single pixels in the ventral-dorsal (V-D) and rostro-caudal (R-C) directions for a single slice. Rat IC (P26). The numbers one to five correspond to the respective areas in each region as highlighted in Figure 10. The 16 single pixels displayed in each region are the same ones used for multi-pixel analysis. **(B)** Mean single-peak amplitudes in each direction as indicated (18 slices; SEM.). **(C)** Mean multi-pixel peak amplitudes in each direction as indicated (18 slices; SEM.). **(D)** Multi-pixel peak amplitudes as a function of the average single pixel peak amplitude in the dorso-ventral and rostro-caudal directions. The second and fourth data points in the dorso-ventral plot overlap. Averages of 18 slices. Error bars are SEM.

**Figure 12:**
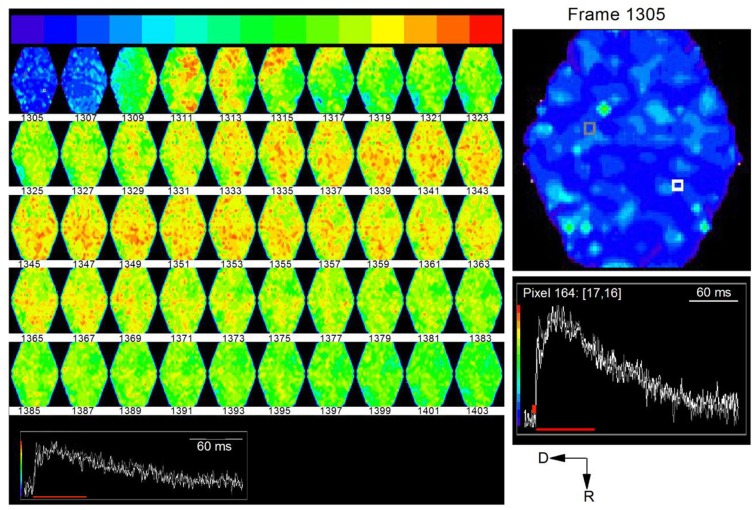
**Time lapse series of the response to a single lemniscal shock in a laminar slice**. Images are 1.22 ms apart. A noticeable response begins in the ventral region of the ICC in frame 1309 (top row: third frame) and propagates dorsally. Inset, white and gray traces correspond to white and gray pixels in the first frame (1305). Red line, region of the response covered by frames 1305–1403. To utilize the full range of colors, the baseline that precedes the response has been set to blue and the peak of the response to red. Right panel, Expanded image of the first frame, 1305, with inset traces of corresponding to the white and gray pixels.

**Figure 13:**
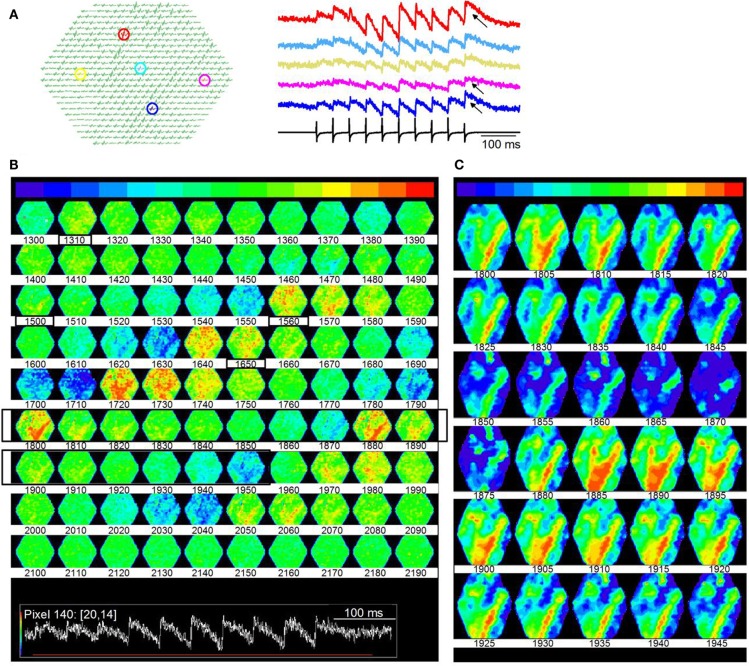
**Microdomains of activity within an ICC lamina**. **(A)** Responses to a 20 Hz LL stimulus train. Shock strength is the same as in Figure 10. Same slice as Figure 10. Left panel, highlighted pixels (arrows); Right panel, corresponding traces; Arrows, regions of accumulating depolarization. **(B)** Time lapse images of responses during the train. Images are 10 frames apart (6 ms inter-frame interval). Frame numbers, Row 1, 1300–1390; Row 2, 1400–1490; Row 3, 1500–1590; Row 4, 1600–1690; Row 5, 1700–1790; Row 6, 1800–1890; Row 7, 1900–1990; Row 8, 2000–2090; Row 9, 2100–2190. Black boxed region, frames 1800–1850 that are illustrated in **(C)**. Inset: Red line, region of the response covered by frames 1300–2190. **(C)** Expansion of black boxed region (frames 1800–1845) in **(B)**. Frames are 3 ms apart. The baseline color in **(C)** has been changed from green to blue to extend the minimum to maximum color scale.

We first briefly describe qualitative features of VSD responses in different regions of the IC in transverse slices (*n* = 86 slices). Slices were oriented to allow visualization, through the 5× objective, of the ICC, the external cortex and the region toward, but not including, the commissure (Figure 3A). We evoked synaptic activity by stimulating LL afferents, and recorded changes in VSD absorbance within the ipsilateral IC. In most regions of the ICC, low stimulus currents (<0.2 mA) evoked net depolarizing responses (Figure 3A, e.g., light blue, yellow, dark blue pixels; Figure 3B). Onset latencies and response characteristics within the ICC are described in detail in Figures 4–13. Outside the ICC, responses in the external cortex were predominantly depolarizing (Figure 3A, red pixel) and prolonged (Figure 3B), with durations of 278 ± 92 ms and average onset latencies delayed by 0.35 ± 0.05 ms compared to those in the ventral ICC (see Materials and Methods). In the commissural region, responses were less frequent (Figure 3A, pink pixel; Figure 3B); average response onsets lagged by 4.22 ± 0.07 ms from the ventral ICC.

Hyperpolarizing (Figure 3A, arrowheads; Figure 3C) or mixed de- and hyperpolarizing (Figure 3A; asterisks; Figure 3C) responses were less frequent than net depolarizing responses. When the net response in a pixel was hyperpolarizing, latencies of the hyperpolarizing peak were rapid (7.8 ± 2.4 ms; 108 events/18 slices) compared to latencies of hyperpolarizing responses that followed a depolarization (34 ± 12 ms; 94 events/27 slices) (Figure 2C; e.g., top and bottom traces). Because the VSD signal measures the summed response of cells within the pixel, a net depolarizing or hyperpolarizing response suggests the direction of the predominant response within that pixel. Thus, with low LL stimulus strengths, we were able to isolate pixels containing groups of cells with a dominance of either depolarization or hyperpolarization. Of the total number of pixels covering the ICC, 78% were depolarizing, 8% were hyperpolarizing, and 14% had mixed de- and hyperpolarizing responses (*n* = 8030 pixels; 32 transverse slices).

A predominantly de- or hyperpolarizing response in any given pixel suggests the direction of the largest response within the population of neurons represented by the pixel. A net depolarizing response within a single pixel is expected from the relatively larger amplitude of excitatory postsynaptic potentials compared with inhibitory synaptic potentials in the ICC (Sivaramakrishnan et al., 2004; Sivaramakrishnan and Oliver, 2006). Pixels with predominantly hyperpolarizing or mixed de- and hyperpolarizing responses were surprising, and suggested small regions where inhibitory strength was unusually large. The area of tissue covered by a single pixel depends on the magnification of the objective lens used. With a 5× objective, each photodetector receives light from ∼17,660 μm^2^ of tissue (150 μm × 150 μm) (J. Y. Wu, personal communication). In a 150 μm thick slice, with a 5× objective, each photodetector (pixel) would record responses from 26,50,000 μm^3^; assuming a total IC volume of 3.8 mm^3^ (Bruckner and Rubsamen, 1995) and a cell count of 3,73,600 (Kulesza et al., 2002), a single pixel (at 5×) represents ∼260 soma, in addition to neuropil. With the 5× objective, these neuronal groups are separated by 150 μm, the inter-pixel distance. Because the VSD signal is proportional to membrane surface area (Xu and Loew, 2003), response variations between pixels could reflect variations in somatic, dendritic, and axonal contributions to the signal.

#### Response properties within frequency regions of the IC

We used VSDs to understand the relationship between responses within the broader topographical regions of the ICC (Merzenich and Reid, 1974; Glendenning et al., 1992; Loftus et al., 2004) and those due to the fine structure of localized afferent inputs within each region (Oliver et al., 2003). In the transverse slice, topographic regions were broadly defined as ventral, medial, and dorsal areas (Figure 4Ai). To determine whether responses in broad topographic regions were scaled responses of smaller areas within each region, we stimulated LL afferents with very low currents (<0.2 mA, 0.1–0.2 ms) to prevent spikes from being generated in single pixels (as in Figure 2E, for example) or the complex oscillations that occur at high activity levels (unpublished observations). We then summed responses contained within a 4 × 4 area covering 16 diodes within each topographic region. The summed multi-pixel response within each region was then compared to the average response of the same 16 single pixels used for summation (see Materials and Methods). This method of analysis treats each broad topographic region as a single compartment, and does not attempt to examine gradients within each region. We first separately describe multi- and single pixel response characteristics, and then compare them. Data are reported from 26 transverse slices (416 pixels in each topographic region).

Summed multi-pixel depolarizations were complex, with multiple peaks and varying magnitudes (Figure 4Aii). Maximum response amplitudes were largest in the ventral ICC, declining in the dorsal and further in the medial ICC. Responses in the ventral and dorsal ICC exhibited at least three clear peaks; multiple peaks were less obvious in the medial ICC, where response magnitudes were lower (Figure 4Aiii, arrowheads). To control for an arbitrary effect of pixel location on response profiles, we compared responses in other 16-pixel regions within the ventral, medial, and dorsal ICC. Figure 4B shows one example of response patterns evoked by 16 pixels that were not in a 4 × 4 grid (Figure 4Bi). Response profiles exhibited the same directional decrease in peak height [ventral to dorsal to medial] (Figure 4Bii) to those in the 4 × 4 grid, as well as the presence of multiple peaks (Figure 4Biii). Additional peaks were observed in several slices with either the 4 × 4 grid or other patterns of clustered pixels (e.g., Figure 4Biii; the response in the ventral ICC has a fourth peak), but did not occur consistently. These results suggested that minimal LL afferent input evoked distinct responses in topographically distinct areas of the ICC.

To quantify differences in the responses between the topographic regions, we used two measures of response magnitude – the maximum response amplitude and the response duration; and two temporal measures – onset latency and the latencies of multiple peaks (Figure 4C). Multi-pixel amplitudes declined from the ventral to dorsal to medial ICC in a 1:0.6:0.3 ratio [*F*(2, 75) = 7.93; *p* < 0.05]. Response durations, which increased from the ventral to medial to dorsal ICC, were also significantly different between the frequency regions [*F*(2, 75) = 3.8; *p* < 0.05], with a ∼15 ms range between the ventral and dorsal ICC. Onset latencies increased systematically from the ventral to medial to dorsal regions, with a ventral-dorsal delay of ∼3.5 ms, suggesting that differences in arrival times of LL inputs in different regions of the ICC was the primary contributor to onset latency. Onset latencies were longer in slices where pixel clusters in the dorsal ICC were chosen at the edge of the slice (8.8 ± 1.6 ms; *n* = 16 slices).

The latencies of the three response peaks exhibited a complex profile. The first peak occurred at approximately the same time in different ICC regions [∼1.1 ms range; *F*(2, 75) = 1.37; *p* = 0.08], suggesting that the initial rise of the summed multi-pixel depolarization was due to either spikes or summation of synaptic responses with little temporal jitter. The frame rate of the VSD camera, ∼1600 Hz (0.614 ms/point), is too slow to record the complete waveform of single sodium-dependent spikes. The presence of a spike-like rapid rise time in the depolarizing waveform seen with multi-pixel measurements is more likely to imply summation, with a slight temporal staggering, of dendritic and somatic synaptic responses with some spike inclusion over the area covered by the photodetectors. The latencies of the second and third peaks, which were greatly delayed compared to the first peak, were clearly graded, with a ventral-dorsal gradient of ∼4 and ∼8 ms respectively, suggesting that synaptic responses formed the primary components of these later peaks.

We next examined responses in the same 16 single pixels used for multi-pixel analysis in each topographic region. In stark contrast to multi-pixel responses, the average single pixel response magnitude and duration did not vary topographically. In all three frequency regions, single pixel responses exhibited a single depolarizing peak (Figures 5Ai,ii); an occasional second peak that did not occur consistently between trials suggested spontaneous activity (Figure 5Ai, asterisk). The magnitude of depolarizations in the ventral, dorsal, and medial regions were the same fractional component [0.2:0.2:0.2; *F*(2, 75) = 0.42; *p* = 0.2] of the multi-pixel response in the ventral ICC. Response durations (measured at half-peak height) did not differ significantly between topographic regions and were within a narrow ∼1.5 ms range [*F*(2, 75) = 1.08; *p* = 0.1]. The similarity in response durations and peak amplitudes between individual pixels suggested that, within small discrete populations of soma, with their accompanying axonal and dendritic processes and LL inputs, the strength of the response (amplitude and duration) was “homogeneous” within a greater part of the ICC, provided the shock strength delivered to the LL was low. These data therefore suggested the presence of response areas of constant magnitude (amplitude and duration) of depolarization, separated by ≤150 μm (the inter-pixel distance at 5×).

Single pixel onset latencies increased systematically from the ventral to medial to dorsal ICC, as they did with multi-pixel measures, providing supporting evidence for their origin from LL afferent input. In contrast to the unchanged response magnitudes and durations, the range of latencies of the peak response was very large between the ventral and dorsal ICC [∼12 ms; 5.2–17.1 ms between the ventral and dorsal ICC; *F*(2, 75) = 10.6; *p* < 0.05] (Figure 5B, bottom right panel). These long latencies suggested the predominance of synaptic responses over spikes, which would be expected from the low LL shock strengths used, which were adjusted to keep single pixel responses sub-threshold.

To examine how single pixel responses translated into the topographical differences in multi-pixel responses, we plotted multi-pixel response measures as a function of the corresponding single pixel measures. Differences in maximum response magnitudes and durations within each broad topographic region (multi-pixel measures) did not follow the same trend as the corresponding single pixel values. First, although single pixel magnitudes did not vary between topographic regions, multi-pixel response magnitudes decreased from the ventral to dorsal to medial ICC (Figure 6Ai). Second, the average single pixel response duration also did not vary between topographic regions, however, multi-pixel response durations decreased from the dorsal to medial to ventral ICC (Figure 6Aii). Thus, the two individual features of multi-pixel response magnitudes, peak amplitude, and duration, had different topographic gradients, although the corresponding single-pixel parameters did not vary topographically. The product of the multi-pixel response amplitude and duration exhibited a slight dependence on the single-pixel product; it decreased from the ventral to medial [*t*(21) = 3.13; *p* < 0.05], and dorsal to medial [*t*(21) = 3.65; *p* < 0.05], ICC, but the gradient between the ventral and dorsal ICC was not significant [Figure 6Aiii; *t*(21) = 1.76; *p* = 0.09]. Taken together, these data suggested that the average response magnitude within single pixels, which was uniform across topographic regions, did not completely account for the broader topographic gradient of response magnitude.

We examined the possibility that temporal summation of single pixel responses gave rise to the topographical (multi-pixel) gradient in response amplitudes. The latencies of the first, second, and third multi-pixel peaks varied linearly with the average latency of the single pixel peak within each topographic region (Figure 6Bi). However, as multi-pixel peak latency increased (i.e., from *p*_1_ to *p*_3_), the topographical differential between single- and multi-pixel peak times also increased. The ventral to medial to dorsal differential increased seven times between *p*_1_ and *p*_2_ (an increase in slope from 0.08 to 0.65) and 0.5 times between *p*_2_ and *p*_3_ (an increase in slope from 0.65 to 0.97). The increase in slope suggested an increased scatter in the times at which responses in single pixels reached their peaks. Figure 6Bii illustrates peak scatter within the group of 16 single pixels in each topographic region in one transverse slice; peak times exhibited the greatest scatter in the medial ICC, and the least scatter in the ventral ICC. Population analysis of the 16-pixel groups in different slices confirmed an increasing gradient of peak scatter in single pixel peak latencies from the ventral to dorsal to medial ICC [14 slices, mean and SD; *F*(2, 39) = 8.58; *p* < 0.05] (Figure 6Biii). The direction of this gradient was similar to the topographical gradient of response magnitudes (compare Figure 4C, top left panel). The relationship between multi-peak response magnitudes and the standard deviation of single-peak latencies was linear, with a slope of −0.43 (Figure 6C; *n* = 14 slices). These results suggested that temporal summation of single pixel responses, which would become more efficient as the scatter of peak times decreased, is likely to contribute strongly to the topographical gradient in response magnitudes.

The comparison between multi- and single pixel response characteristics thus suggests the presence of unitary areas of response magnitude in the ICC that sum temporally to create a graded response magnitude that decreases from the ventral to dorsal to medial ICC.

To examine the spread of activity within and across frequency regions in the transverse slice, we plotted time-lapse images during the response to a single LL shock under low- and high-gain to visualize different spatial features (Figure 7; frame intervals are one frame; 0.6 ms apart). Regions in the ventral and dorsal areas (Figure 7A, white and gray pixels, frame 376; traces, inset at bottom; expanded in the right panel) were chosen to illustrate examples of responses. At low-gain, activity was observed primarily in three segregated bands. A band that began initially in the ventral region (frame 378), propagated into the central ICC within ∼2.4 ms (frame 382). The origin of this ventral band, close to the entry of LL fibers, its propagation perpendicular to the direction of the frequency laminae, and the short propagation time, suggested primary propagation along lemniscal input tracts. A second narrower band of activity, first appearing in the dorso-lateral region (frame 379) and propagating dorso-medially, reached its maximum spatial extent in ∼3 ms (frame 384) and is likely to reflect propagation along the low frequency laminar region of the IC. A third band of activity, in the region of the external cortex, which began with the initial spread of lemniscal inputs in the ventral IC (frame 393), remained after both the ventral and dorsal band had declined (up to frame 415), reflecting prolonged responses in the external cortex (Movie S1 in Supplementary Material).

Images analyzed at higher gains revealed possible cross-laminar propagation (Figure 7B). In addition to the three primary regions of activity seen in the low-gain images, now clearer at slightly earlier times (frame 378), activity in a direction perpendicular to or angled to the main three bands was clear during the later part of the response (e.g., frames 401–415). In frame 401, for example, this “cross-laminar” movement began at the medial end of the dorsal band and, in successive frames, propagated ventrally and then back into the dorsal band. This pattern of movement from the dorsal or ventral laminae into the central portion of the ICC, and the consequent formation of additional activity bands was a consistent finding in all transverse slices (*n* = 56) in which either a strong dorsal or strong ventral band was present, suggesting that the relatively weaker strength of these additional bands did not necessarily arise from cut connections in any particular slice. The precise positions of bands and their widths varied between slices, sometimes occurring more medially or more laterally, and orientation angles differed slightly, which would be expected from variations in the slice plane, the number of intact connections, and the currents used to activate LL fibers.

#### Propagation of activity across the commissure

Cross-talk between the two inferior colliculi through the commissure regulates the balance of excitation and inhibition in the IC (Moore et al., 1998; Ingham and McAlpine, 2005; Malmierca et al., 2009). Here we use VSDs to show that blocking GABA_A_-mediated inhibition following activation of ipsilateral LL inputs causes excitation to propagate through the commissure to the contralateral IC, suggesting that functional ipsilateral-contralateral (excitatory) connectivity requires an excitation threshold.

To record the effects of GABA_A_ antagonists on commissural propagation, we first set a low level of baseline excitation in normal ACSF to prevent the onset of depolarization block in the presence of GABA_A_ antagonists, both in brain slices and *in vivo*, when normal excitation levels are already high (Sivaramakrishnan et al., 2004). Excitation was kept low by stimulating the LL with low-frequency shock trains, and adjusting the current strength to generate weak depolarizing responses that further depressed during the train. With a 20 Hz train (0.2 mA shock strength), for example, depolarizing potentials were restricted to the ICC, with little or no commissural activity (Figure 8A, left panel). Response magnitudes, illustrated in individual pixels, did not differ greatly in different regions of the ICC (e.g., Figure 8A, right panel, pink and red traces), however they declined toward the commissure (e.g., Figure 8A, right panel, light blue and yellow traces). In the presence of the GABA_A_ antagonist SR95531 (20 μM), activity during the train increased in the ICC and depolarizing responses were now observed in the commissural region (Figure 8B, left panel). In the ICC, responses to the individual shocks in the stimulus train increased in amplitude, they no longer depressed and were oscillatory (Figure 8B, right panel, pink and red traces). Spikes now occurred in the commissural region with non-linearities suggestive of spike collisions (Huang et al., 2010) (Figure 8B, right panel, yellow and blue traces; boxed region).

To quantify the effects of SR95531 on ICC and commissural activity, we averaged responses separately over a large part of the respective regions (Figure 8Ci, top panels, red and blue pixels). In control conditions, the 20 Hz LL stimulus train evoked a depressing but stimulus-locked response in the ICC (Figure 8Ci, bottom left panel; red dotted lines on traces), with no measureable activity through the commissure. In SR95531, responses in the ICC remained stimulus-locked (Figure 8Ci, bottom right panel; red dotted lines on traces), however, they exhibited oscillations, and prolonged responses that arise from synchronization (SS, unpublished observations). In addition, responses facilitated. The facilitation/depression index (Sivaramakrishnan et al., 1991) of the responses to the first and last pulses in the train [(VSD_10_ − VSD_1_)/VSD_1_] showed a 53% depression at the last pulse of the train compared to the response to the first pulse in control conditions (Figure 8Cii). In SR95531, the loss of depression did not result in a return to steady excitation (a depression/facilitation index of zero), but instead facilitated by almost 85% compared to the first pulse in the train, a 30% increase in response amplitude compared to the control [19 slices; *t*(18) = 3.84; *p* < 0.05].

The commissural region showed clear evidence of spikes in SR95531, suggesting a high incidence of synchronous firing in commissural axons (Figure 8Ci, bottom right panel). Importantly, however, whereas responses were stimulus-locked during the early part of the train, large oscillations caused temporal smearing toward the end of the train (Figure 8Ci, bottom right panel; red dotted lines on traces). Responses outlasted the stimulus by several hundreds of milliseconds (300–950 ms across 12 slices) and exhibited different degrees of oscillatory behavior. The change from spikes and stimulus-locked responses during the early part of the stimulus train, toward a greater prevalence of oscillations and decreased incidence of stimulus locking toward the later part of the train suggested a time-dependent increase in activity in the commissure. In control conditions, commissural activity did not change during the early part of the train [Figure 8Ciii; 200 and 400 ms time points; *t*(18) = 0.98; *p* = 0.3], but increased slightly toward the end of the train [50% increase; *t*(18) = 3.61; *p* < 0.05; 19 slices]. In SR95531, a steep increase during the earlier part of the stimulus saturated at ∼400% of the control toward the later part of the train (*n* = 19 slices).

Although the incidence of commissural propagation in the absence of GABA_A_ antagonists was rare with low frequency LL stimulus trains, very high-frequency (>80 Hz) trains did evoke prolonged commissural excitation (14 slices, data not illustrated), suggesting that propagation of excitation through the commissure to the contralateral IC requires an excitation threshold in the ipsilateral ICC. Reducing inhibition is one way of reaching this threshold, and may involve neuronal circuitry.

Time-lapse images illustrated oscillatory response patterns during the latter part of the train that coincided with commissural propagation. To prevent the normal spread of activity within the IC from masking the direction of commissural propagation, we filtered out low excitation levels and restricted analysis to the region of peak depolarization, which was scaled to be within the light green-red color range (Figure 9A, inset traces; frame intervals are 42 frames; 25 ms apart). In normal ACSF, the depressing peak excitation during the train is illustrated in the nine frames that correlate with each shock in the train (Figure 9A; frames 942, 1026, 1110, 1194, 1278, 1362, 1446, 1530, 1614). With this analysis window, the strongest excitation was restricted to the ventral ICC where the lemniscal afferents enter; there was little spread into the central ICC and no evidence of commissural activity. In the presence of SR95531, the strongest activity remained temporally locked to each shock in the train, occurring approximately at the same frame times as the control. Activity remained restricted mainly to the ventral region of the ICC, although there was a greater spread at some times during the train (Figure 9B, right panel; e.g., frames 942, 1278), and less depression. The main features of interest, however, are frames 1530–1614, which correlate with the region of non-linear responses in traces from the commissural region (Figure 8B, boxed region). Activity in some of these frames did not coincide with a shock in the train (Figure 9B; e.g., commissural propagation in frame 1572 occurred between the eighth and ninth shock of the train), a contrast to the clear 1–1 stimulus-response correlation in normal conditions (Figure 9A; frame 1572, during which there was no LL shock, did not show a response) (Movies S2 and S3 in Supplementary Material).

### Activity in the intra-laminar plane of the ICC

To examine connections within a lamina, we previously developed a laminar slice plane of the ICC that preserves intra-laminar connections and minimizes inter-laminar circuitry (Sivaramakrishnan and Oliver, 2006; Figure 1B). By limiting the thickness of the slice to 100–150 μm, the extent of laminar spread (Schreiner and Langner, 1997), the number of laminar planes is theoretically restricted to a single “sheet,” with highly reduced circuitry that contains a laminar module with its fibro-dendritic arrangement of input lemniscal fibers traversing the central nucleus parallel to and synapsing in the dendritic fields of disk-shaped neurons (Oliver, 2000; Malmierca et al., 2005). The laminar slice plane contains only one colliculus, primarily the ICC, and intact LL afferents. Whole-cell recordings from neurons in this slice plane demonstrate an extensive stimulus-dependent polysynaptic influence on response properties (Sivaramakrishnan and Oliver, 2006), suggesting that local interactions within a lamina increase with afferent recruitment.

As with the transverse slice, stimulating electrodes were placed on the LL before it entered the IC, and a field electrode, *f*, recorded the stimulus (Figure 10A). To increase spatial resolution, we used a 20× objective instead of the 5× used with the transverse slice. Each pixel would cover responses from ∼65 neurons (compared to ∼260 neurons with the 5× objective), with a pixel separation of 37 μm. With the 20× objective, we isolated a small portion of the lamina for image acquisition and analysis (Figure 10A; boxed area). We illustrate responses within the boxed region following activation of the LL with a single shock (Figures 10–12) and a 20 Hz train of 10 shocks at the same current strength as the single shock (Figure 13). Single shock results are reported from 18 laminar slices and train results from four additional (22) slices.

#### Responses to a single shock

Responses evoked by a single LL shock were dispersed within a lamina. We measured the spatial response profile by summing responses over multiple pixels within circumscribed areas progressively (1–5) in either the ventral to dorsal (VD) (Figure 10Bi) or rostral to caudal (RC) (Figure 10Ci) directions. Each of these multi-pixel areas was treated as single compartment. In both the VD and RC directions, depolarizations were widespread and generally contained an initial spike followed by a slower synaptic response (Figures 10Bii,Cii, single and double asterisks). We did not observe the complex multi-peaked responses seen in transverse IC slices under similar stimulus conditions. To compare differences in propagation between the VD and RC directions, we used measures of the latency of response onset and the peaks of the spike and synaptic responses, measured from the onset. We measured all times with respect to the onset latency at either the ventral or the rostral end of the portion of the ICC in the image field.

Two key differences in peak times between the VD and RC directions suggested both temporal inhomogeneities as well as homeostasis within the lamina. First, onset latencies were identical along the VD axis [*F*(4, 65) = 0.8; *p* = 0.9], but increased along the RC axis with a range of ∼0.65 ms [Figure 10D, top; *F*(4, 65) = 15.7; *p* < 0.001; *n* = 14 slices], suggesting an axis-dependent functional orientation of LL axon path length. Second, the gradient of spike peak times was larger in the VD direction [Figure 10D, middle panel; RC gradient 0.6 ms; VD gradient 3.1 ms; *t*(13) = 4.22; *p* < 0.05], however, the time to peak of the synaptic response showed no gradient in either direction [Figure 10D, bottom; VD, *t*(13) = 1.28; *p* = 0.2; RC, *t*(13) = 1.14; *p* = 0.27]. Although a gradient in synaptic latency was absent along either axis, absolute latencies were axis-dependent, being longer by ∼6 ms in the RC direction [Figure 10D, bottom panel; *t*(13) = 5.51; *p* < 0.05]. A gradient in the latency of the spike but not the synaptic response suggested that secondary local interactions within the lamina counteracted the temporal gradient caused by afferent input, and exerted homeostatic control of synaptic peak latency. Homeostasis could occur through differences in membrane time constants (Sivaramakrishnan and Oliver, 2006) or synapse location on dendrites.

Single pixel responses consisted mainly of synaptic activity, with no detectable evidence of the spike observed in the multi-pixel summed responses (Figure 11A), which was expected from the low LL stimulus shock strength which was adjusted to keep single pixel responses sub-threshold. The presence of spikes in multi-pixel responses could arise from summating local interactions between single-pixel response areas. Responses in single pixels had similar maximum amplitudes along both axes [Figure 11B; VD, *F*(4, 85) = 1.55; *p* = < 0.05; RC, *F*(4, 85) = 2.05; *p* < 0.05]. These response areas were 37 μm apart (the inter-pixel distance with the 20×), closer than the 150 μm separation in the transverse slice.

Multi-pixel responses, measured over each of the broad areas (1–5) and normalized to responses in either the ventral or rostral regions respectively, also had uniform response amplitudes without significant gradients in either the VD or RC directions [Figure 11C; VD, *F*(4, 85) = 0.8; *p* < 0.05; RC, *F*(4, 85) = 1.01; *p* < 0.05]. A comparison of multi- and single pixel measures of maximum synaptic response magnitude indicated that the transfer function between single- and multi-pixel magnitudes was also not graded in either the VD or RC direction, with no scaling of response magnitude [Figure 11D; VD, *F*(4, 85) = 1.3; *p* < 0.05; RC, *F*(4, 85) = 1.26; *p* < 0.05; *n* = 18 slices].

Time-lapse images of responses to the single shock showed a wave of activity that began at the ventral end and propagated dorsally and rostrally (Figure 12, e.g., frames 1307, 1309; frame intervals are two frames; 1.2 ms apart). This initial wave corresponded to the first peak of the depolarizing response caused by LL axonal spikes. The secondary activity that followed this initial wave was widely distributed in the region of the ICC being imaged. This second phase corresponded temporally to the slower phase of the depolarizing response, resulting mainly from synaptic potentials and probable cellular spiking. The directional nature of LL afferent input and its restricted spatial spread, followed by the distributed nature of the synaptic response, suggests widespread effects within a lamina from a low level of afferent input (Movie S4 in Supplementary Material).

#### Responses to a shock train

In contrast to the dispersed activity evoked by a single LL shock, stimulus trains re-organized activity in the lamina into distinct spatial domains, suggesting that local circuitry within a lamina creates spatio-temporal response compartments. Shock strengths of each stimulus in the LL train (20 Hz stimulus frequency) were delivered at the same current strength as the single shock described in Figure 10. Each shock in the train evoked a depolarization (Figure 13A). Unlike the uniformity of response amplitudes observed with the single shock, responses in different regions of the ICC varied in amplitude and the degree of accumulating depolarization (Figure 13A, right panel, arrows) during the train. Distributed responses, similar to those evoked by single shocks did occur during the train, but only to the first few stimulus pulses (∼120 ms from train onset) (Figure 13B; frames 1310–1500; boxed frame numbers rows 1 and 3; frame intervals are 10 frames; 6 ms apart). Corresponding to the region of accumulating depolarization, response magnitudes increased greatly (e.g., Figure 13B, frames 1560–1650; boxed frames numbers rows 3 and 4), and organized this region of the ICC into bands of high activity (Figure 13B, frames 1800–2070; rows 6–8).

We plotted time-lapse images of banded activity (Figure 13B; Frames 1800–1950; rows 6, 7; black boxed regions) with greater temporal resolution (Figure 13C; 3 ms between frames). In this particular slice, the banded region began ∼300 ms (frame 1800) after the onset of the train (frame 300), when depolarization began to accumulate (as in Figure 13A, traces). The pattern consisted of the primary band and secondary orthogonal bands running oblique to either the dorsal-ventral or rostral-caudal axes. In other laminar slices where similar minimal stimulation was used (*n* = 22), bands were oriented differently, slightly deviating from the angles described in this figure, which is expected if a particular band depends on the subset of lemniscal afferents activated. 5/22 slices exhibited a single band oblique to the dorso-ventral axis, 7/22 slices displayed two distinct bands, parallel to the rostro-caudal axis, and 10/22 slices displayed two bands, one of which was parallel to the rostral-caudal axis, the other at an oblique angle to the dorso-ventral axis. The bands remained consistently strong once activated, also suggesting prolonged depolarizing responses. In single ICC neurons, accumulating depolarization results from synaptic summation and changes in intrinsic ion channel activity (Sivaramakrishnan and Oliver, 2006); the spatial information obtained from VSD images suggests that accumulating depolarization recruits a subset of the lamina into regions of high activity.

### Stimulus-and cell type-specific microcircuits in the ICC

**Figure 14:**
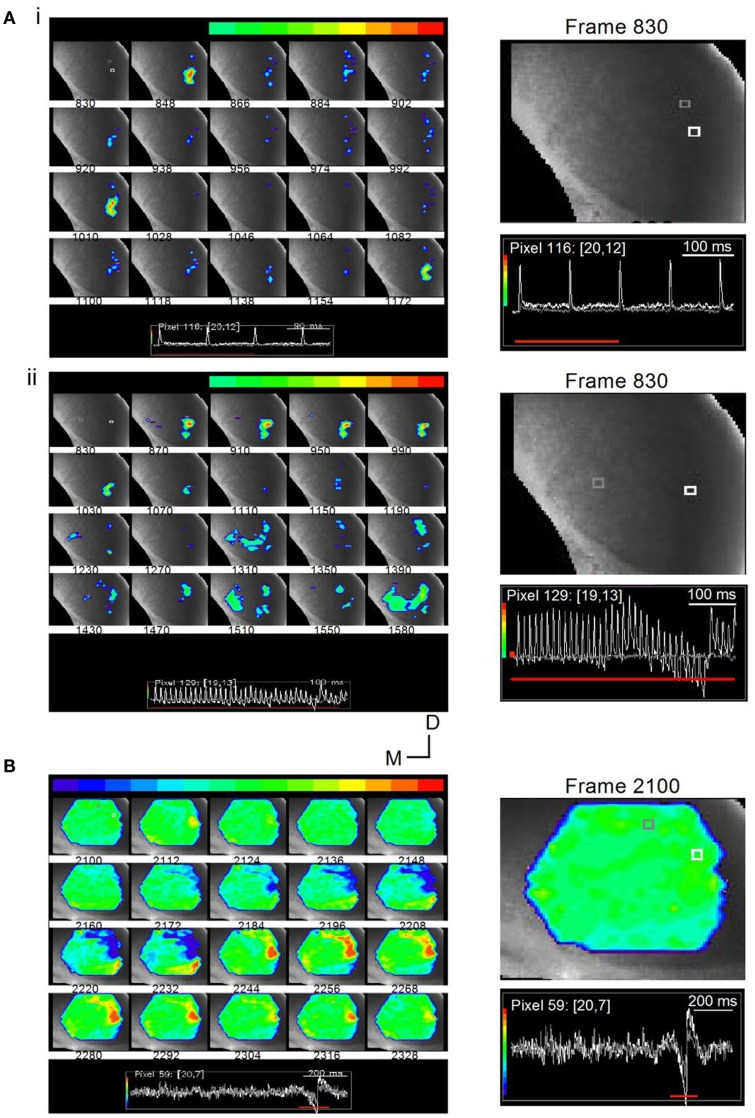
**Stimulus-specific microcircuits in the ICC**. **(A)** Time lapse images of responses in a transverse IC slice (mouse; P20) to changing LL stimulus frequencies. (i) Responses to a 10 Hz train are localized to the ventral ICC where LL axons enter. Frames are 12 ms apart. Inset: White and gray traces correspond to the similarly colored pixels in the first frame 830. The pixels chosen are close together to illustrate the spatially restricted nature of the response. While the response at the white pixel is large, there is no response at the gray pixel. Responses in frames 848, 1010, and 1172 correspond to the times of each of the three shocks (100 ms apart). Right panel, Expanded image of the first frame, 830, with inset traces of responses in white and gray pixels; Red line, response area (three of the four shocks) covered by frames 830-1172. (ii) Responses to a 40 Hz train spread and exhibit accumulating non-linearities. Shock current strength is the same as in (i). Frames are 25 ms apart (the same separation as the interpulse interval). The 1–1 stimulus-response coupling as well as the spatially restricted response seen in the early frames (870–1070) is lost during the later part of the train (frames 1310–1590). Inset: Color range is chosen to highlight the depolarizing response. Instances of hyperpolarization, e.g., activity below baseline seen toward the end of the train, are therefore omitted from the total response; Right panel, Expanded image of the first frame, 830, with inset traces of responses in white and gray pixels. **(B)** Time lapse images of responses in a transverse IC slice (rat; P25) to a 1-s long train at 40 Hz. Shock strength 0.15 mA. Frames are 7.2 ms apart. Response region (frames 2100-2328) corresponds to the inhibitory and excitatory motifs of the rebound region only (inset, red line). Color scale is adjusted so that the baseline is set to green to allow for inhibitory responses to appear toward the blue regions. Frame 2112 shows a small localized depolarizing response. This frame precedes the onset of inhibition (frame 2148) by 22 ms. Directional scale bar applies to **(A,B)**. Right panel, Expanded image of the first frame, 2100, with inset traces of responses in white and gray pixels.

Whole-cell patch recordings of LL-evoked responses in single ICC neurons suggest that the amplitude and duration of synaptic potentials, the frequency of spiking and the ability to follow their inputs is determined by the relative numbers and frequency of afferent axons activated, which can be varied by changing the strength and frequency of electrical shocks applied to the LL (Sivaramakrishnan et al., 2004). We now use VSD imaging to highlight the importance of stimulus parameters in determining the specificity of functionally active circuits in the ICC. We illustrate the stimulus-circuit dependence using two LL stimulus paradigms: (1) low- and high-frequency trains that produce different spatio-temporal profiles (Figure 14A), and (2) a long train at very low input strength that isolates a cell-type specific local circuit (Figure 14B).

With a low frequency, 10 Hz train, responses were locked to each stimulus (Figure 14Ai, frames 846, 1010, 1172; frame intervals are 18 frames; 11 ms apart), were of equal magnitude, decayed rapidly, and were confined to the ventral region of the ICC. This response can be explained as a relatively simple recruitment of a small, localized, population of cells close to the region of LL entry in the ICC. In the same slice, when the train frequency was increased to 40 Hz, the activity profile indicated a spatial recruitment of other regions in the ICC and, in addition, a temporal component to the recruitment. Stimulus-response locking occurred during the first several stimuli, with spatially restricted, rapidly decaying responses (Figure 14Aii, frames 870–1150; frame intervals are 40 frames; 24 ms apart), however, ∼300 ms after the train onset, responses spread from the LL entry point, laterally, medially, and dorsally (frames 1310–1590). Thus afferent input frequency may be a factor in the recruitment of previously silent microcircuits in the ICC (Movie S5 in Supplementary Material).

The second stimulus pattern, which is especially interesting in its ability to isolate a circuit that is specific for a certain physiologically defined cell-type is a long, low frequency train that results in a gradual build-up of inhibition followed by post-inhibitory rebound firing. Intrinsic rebound neurons comprise almost 50% of the physiologically defined cell-types in the ICC (Sivaramakrishnan and Oliver, 2001), and rebound spikes, whether generated intrinsically or through lemniscal stimulation, can be abolished by NiCl_2_, an antagonist of T-type calcium channels (SS unpublished observations). VSD imaging shows that rebound neurons form characteristic microcircuits, defined by a focal point of initiation that then spreads to an annular ring that oscillates between inhibition and excitation. The current strength of the stimulus pulses in the train is adjusted so that little or no depolarizing activity occurs during the train, and the predominant response is the rebound motif, consisting of a hyperpolarization followed by a rebound depolarization, that occurs at the end of the train. 400 ms-2 s long trains were found to be most efficient at evoking a single rebound motif at the end of the train.

In the slice illustrated in Figure 14B, the time lapse images span the region just preceding and covering the rebound activity (bottom, inset, red line). Frame intervals are 12 frames (7.2 ms) apart. The 1500 ms long train evoked minimal depolarization, apparent as a small, localized region of activity in the ventral ICC (frame 2112), just preceding the onset of the rebound motif. Inhibition that preceded the rebound depolarization was observed in the ventral ICC spreading toward the lateral edge (frame 2160), increasing in strength and area in the succeeding frames. A focal point of depolarization then began (frame 2198), increased in strength and spatial spread, and covered most of the area that was previously inhibitory (frames 2224–2280). Within this region that was now excitatory, an annular ring developed (frames 2244–2280), which corresponded to the excitatory region of the rebound motif, and was followed by a gradual fading of response in the succeeding frames. (Movie S6 in Supplementary Material). Rebound motifs occurred in different areas of the slice; the sequence of inhibition, focal excitation, and annular ring of depolarization was characteristic (32 slices). The observation that the same or similar area switches between excitation and inhibition, as well as the loss of the pattern in the presence of NiCl_2_ (data not shown), suggests that the rebound motif occurs in cells with an intrinsic rebound firing pattern.

These results suggest the activation of local circuits in the ICC from interactions between synaptic and intrinsic membrane parameters. The spatial and temporal spread of activity that occurs when LL activation switches from a low to a high-frequency train suggests the recruitment of large cell populations that create a network through which activity propagates out into the greater part of the ICC. The establishment of these local networks, which require a change in synaptic strength, is likely to include cells that differ intrinsically. On the other hand, local circuits involving post-inhibitory rebound cells form a loop of alternating excitation and inhibition, restricted to the same spatial region. Within this region, cells interconnect to form these alternating patterns, thus the network arises when activity propagates recurrently between these interconnected cells. There is little spread of activity beyond this region of alternating excitation and inhibition, however, suggesting that rebound firing recruits regions of the ICC into closed-loop local circuits.

## Discussion

We describe optical techniques using VSDs to measure activity within and across frequency laminae and to isolate stimulus- and cell-type-specific circuitry in the IC. The central nucleus of the IC appears to operate from a base of functional units that are homogenous in response magnitude, but graded in response time. As input parameters change, these units interact to create a global activity pattern that is directionally sensitive, topographically from high to low to middle frequencies, or along the rostro-caudal or dorso-ventral axes within frequency laminae. Our results suggest the presence of an intrinsic spatial activity profile in the central nucleus created by local interactions between small populations of temporally graded, equal magnitude, response areas.

### Focal versus global activity and the concept of independent response areas in the ICC

The comparison of VSD responses in single pixels with those in a broader area covered by multiple pixels attempts to address the general question of whether responses in small subsets of ICC neurons scale to produce responses in larger populations. The concept of synaptic domains, consisting of clustered inputs with similar functionality (Loftus et al., 2010) residing within the broader topographical frequency mosaic of the ICC, the gradation of timing information along the medial-lateral (Schreiner and Langner, 1988; Langner et al., 2002), and of spectro-temporal features along the dorso-ventral axes (Rodriguez et al., 2010) and the postulated functionally segregated units for sound frequency (Semple and Aitkin, 1979), all strongly indicate the existence of a response transfer function from sub-domains of neurons into a larger topographical organization. Our VSD data suggests that, under conditions of very low afferent activation, small groups of neurons, together with their inputs, form spatially constricted response areas with uniform magnitude but temporal spread. A differential in the temporal summation of these response areas results in a gradient of response magnitude between the broader topographic regions of the ICC, introducing topographic-dependent gain control.

Population coding in the auditory midbrain has compared single neuron responses with responses pooled from multiple neurons by using average firing rates or spike trains as the measure of activity (Fitzpatrick et al., 1997; Schneider and Woolley, 2010). Since changes in firing rate can arise from changes in either or both the magnitude and timing of the sub-threshold response to inputs, we designed our stimulus paradigms so that responses occurred throughout the ICC and, in individual pixels, were primarily sub-threshold. Together with the use of the low-power objective through which much of the ICC was visible, this stimulus paradigm allowed us to simultaneously measure activity in both large and small neuronal populations evoked by small, “baseline” stimuli that would evoke responses in a greater portion of the ICC.

The uniformity, throughout the ICC, of sub-threshold response magnitude within the small neuronal population represented by a single pixel suggests that, without significant non-linearity in neuronal responses, synaptic strength is constant throughout the ICC. The single-peaked, small duration, depolarizing responses in individual pixels are similar to those obtained with whole-cell patch recordings from single ICC neurons in brain slices using similar minimal LL stimulation (Sivaramakrishnan and Oliver, 2006), thus at these low stimulus levels, the lack of activation of postsynaptic voltage-dependent currents prevents the diversity of ICC intrinsic properties (Sivaramakrishnan and Oliver, 2001) from affecting response patterns. Thus, in the probable absence of active conductances, sub-threshold response magnitudes in the ICC appear as functionally defined response areas.

In contrast to the uniformity in sub-threshold response magnitude across topographic regions, a clear temporal gradient existed between single pixels. This gradient included a slight gradient in onset latency, but predominantly, a gradient in the time of occurrence of the peak depolarization. Thus between each functional module, responses vary either in onset or rise time, and produce a systematically graded effect on the timing of the peak response in the ventral to dorsal, and lateral to medial directions. In the transverse slice, a micro-temporal gradient within a module could occur in the 150 μm rostral-caudal or in the 150 μm × 150 μm dorso-ventral and lateral-medial directions covered by each pixel. This gradient could arise intrinsically through differences in membrane time constants (Sivaramakrishnan and Oliver, 2006), or extrinsically, from different brainstem sources (Schofield, 1991; Gabriele et al., 2000; Saldaña et al., 2009) through lemniscal axons with variable path lengths and diameters. Afferent LL weighting might become more important at higher recruitment levels and create greater variations in single pixel responses within and across topographic regions.

Global activity, in areas selected in transverse ICC sections to analogously represent the topographic distribution of frequency regions, exhibited a gradient in both response magnitude and timing. In contrast to the predominantly sub-threshold activity in single pixels, the summed activity in multiple pixels clearly demonstrated the presence of spikes followed by synaptic responses. In VSD signals, for spikes to become visible as a component distinct from synaptic responses, there must be sufficient synchrony. If the predominance of spikes was due to an increased directional ratio, along a topographic axis, of lemniscal or ICC neuronal axons to soma, then spikes should have become increasingly visible in single pixels in either the ventral-dorsal or lateral-medial directions. Because this did not occur, we conclude that the increased prevalence of spikes in these global regions resulted from the temporal summation of sub-threshold synaptic potentials between neighboring single pixels, suggesting that local circuits exist between functional units, and their activity increases neuronal output. A similar local circuit effect on sub-threshold summation is likely to underlie the increased response amplitude; multiple peaks with varying latencies and the changing response magnitude with direction are likely to result from the temporal gradient between individual pixels. Gain control in the ICC is integral to auditory coding (Palombi and Caspary, 1996; Ingham and McAlpine, 2005). Our VSD data suggests that, under minimal stimulus conditions, gain control occurs through *temporal* enhancement via local circuits, within an effective radius ≥150 μm, the inter-pixel distance. We cannot, however, rule out additional changes in gain within the smaller spatial units imaged by single pixels.

### Circuitry within and across frequency laminae

A comparison of LL-driven activity between the transverse and laminar slice planes suggests an intriguing functional organization of frequency domains. In transverse slices, bands of strong activity appear to occur roughly in the direction of lemniscal input (Saldaña et al., 2009), and might indicate fibro-dendritic laminae. The width or dorso-ventral extents of these bands suggests several overlapping laminae which are not resolvable with low-power objectives. However, we were able to distinguish at least three main regions of lemniscal propagation in the ventral and dorsal ICC and in the external cortex and to measure the time taken by activity to propagate into these regions. The ∼2.5 ms time taken by lemniscal input to reach the dorsal and middle regions of the ICC is a rough estimate of the maximum contribution of travel time in the ICC to the onset latency to sound, which is ∼10–20 ms for most ICC neurons in the unanesthetized mammal (Kuwada et al., 1997; Sivaramakrishnan et al., 2004). A second interesting aspect of the initial strong activity pattern is the response in the external cortex that lasted long after the response in the ICC had declined, suggesting slow processing, a characteristic that may enhance integration of the different sensory inputs to this region (Robards, 1979; Zhou and Shore, 2006). In contrast to the strong activity in the direction of laminae, the cross-laminar connections appeared to be much weaker, and delayed. Thus cross-laminar movement might be a secondary, rather than a direct, consequence of lemniscal input, with boosting by local circuits.

Inhibitory postsynaptic potentials in the ICC evoked by commissural activation show paired-pulse facilitation (Vale and Sanes, 2000), suggesting that the GABergic inhibitory component of commissural connectivity (Hernandez et al., 2006) exerts a non-linear effect on ICC excitation. VSD imaging shows that commissural connectivity is more easily established when GABA_A_ receptors are blocked, providing supporting evidence for the damping effect of the commissure on ICC excitability. The change from a normally depressing to a facilitating excitation in the ICC in the absence of GABAergic activity is likely to result from the loss of facilitating inhibitory synaptic potentials and may contribute to the gain control exerted by inhibition in the ICC (Palombi and Caspary, 1996; Sivaramakrishnan et al., 2004; Ingham and McAlpine, 2005). The shape of spikes in the commissural region, suggestive of collisions, indicates bidirectional activity in the commissural bundle caused by spikes moving in opposite directions in different commissural axons. The gradual change from spikes to collisions and oscillations in the commissure is time-dependent, thus propagation through the commissure contributes to a temporal regulation of the excitatory-inhibitory balance in the ICC. Since the whole slice was bathed in GABA_A_ antagonists, we did not establish whether the increased excitation that triggered commissural propagation was localized to specific ICC regions or arose from the contralateral ICC from similar or dissimilar frequency bands (Malmierca et al., 2009).

Voltage-sensitive dye images of activity within frequency laminae illustrate a complex functional architecture, where directional homeostasis, the maintenance, along the dorso-ventral or rostro-caudal axes, of synaptic latency in spite of a changing input onset latency, appears to be created through a stimulus-dependent recruitment of local circuits. Within a particular direction, homeostasis was maintained in the temporal response of ICC neurons to LL inputs; irrespective of a gradient in lemniscal input times, a gradient in cellular response times did not develop in either direction. Along the rostral-caudal axis, the latencies of response onset and the peak of the first spike both varied, which is expected from the direction of LL afferent input (Saldaña et al., 2009), and their gradients were similar, however, the latency of the synaptic peak showed no gradient at all. Along the ventral-dorsal axis, there was no change in response onset, a larger gradient in first spike latencies compared to the rostral-caudal direction, and yet, no gradient in synaptic peak times. These results suggest an intrinsic regulation of synaptic response gradients within a lamina. The longer absolute synaptic response times in the rostral-caudal direction, cannot be explained by the small range in response onsets or first spike latencies, but could be due to differences in cell diameters, synapse clustering, or input strength. We caution that the homeostatic control of synaptic response time occurs when the entire LL afferent tract is activated by a single shock and future studies in the brain slice will benefit from single LL axon stimulation to isolate specific spatially segregated inputs. In spite of this drawback, however, our results demonstrate that a gradient in input arrival times does not necessarily translate into a gradient in cellular response times between groups of cells spaced 37 μm apart.

Re-orientation of activity within a lamina triggered by LL stimulus trains suggests that intra-laminar connections recruit sub-sets of ICC neurons into spatial patterns of activity. Much of this recruitment is due to the accumulation of depolarization during the train. Plateau or accumulating depolarizations recorded in single ICC neurons in a laminar slice result from polysynaptic inputs suggestive of local circuit activation (Sivaramakrishnan and Oliver, 2006), and VSD images indicate that intra-laminar circuitry creates spatial orientation within laminae. Taken together with the single shock data, these results indicate that the anatomical and physiological axes within ICC frequency laminae (Lim and Anderson, 2007) arise from a local circuit-driven enhancement or suppression of specific sets of input domains.

### Microdomains of local circuits in the ICC

A major advantage of using VSD imaging in the ICC is the ability to image different components of circuitry and therefore design stimuli to activate specific regions of the ICC in a relatively controlled way. We have used two stimulus paradigms to illustrate how responses propagate from a point of origin and reveal functional architecture in the ICC. Low frequency stimulus trains, for example, produce localized responses, a one-to-one stimulus-response relationship and consistency in spatial dimension. The highest activity levels in local circuitry activated by this type of stimulus-response coupling is restricted to near neighbors of ICC neurons, with its spatial extent determined by the stimulus current and the number of LL axons activated. The greater part of the ICC, which remains mainly silent, is recruited into activity by changing the frequency of the stimulus. Localization of the response at the beginning, and its spread only during the latter part of the train, suggest the requirement for a build-up in depolarization before activity can spread into other ICC regions, reinforcing the concept of local circuit activation. The long stimulus train at low current strength that activates circuits involving post-inhibitory rebound cells also has a point of origin from which it propagates. This circuit differs from those activated by the low- and high-frequency trains described above in that it oscillates between inhibitory and excitatory activity. The presence of a clear rebound motif within the single pixel implies that most of the cells within that pixel are post-inhibitory rebound neurons, suggesting that these neurons might be grouping together. This grouping is likely to provide a sufficiently strong point source that begins the propagation into an annular ring of excitatory-inhibitory oscillation.

## Conclusion

Voltage-sensitive dye imaging is a viable tool to correlate activity in small groups of cells with those in larger populations in slices of the IC. The ability to distinguish between spikes and synaptic potentials provides a way of separately analyzing the fast and slow components of a response in any given region. Since spikes do contribute to the total signal, however, there is some smearing of temporal components. VSD imaging of IC activity benefits from multiple approaches to both acquire and analyze optical data. A comparison between single- and multi-pixel summated responses suggests the presence of unitary activity domains and non-linear processes that create spatio-temporal activity profiles. Activity within and across frequency laminae, which appears to have different strengths and a dynamic spatial and temporal spread, can be revealed by using specific stimulus patterns, windowing responses, and varying the image gain to measure activity at the peak and closer to sub-threshold regions of the response. Stimulus patterns derived from knowledge of the electrophysiological behavior of identified cell-types are an especially important tool to establish the presence of cell-type specific microcircuitry in the ICC.

## Conflict of Interest Statement

The authors declare that the research was conducted in the absence of any commercial or financial relationships that could be construed as a potential conflict of interest.

## Supplementary Material

The Supplementary Material for this article can be found online at http://www.frontiersin.org/Neural_Circuits/10.3389/fncir.2013.00041/abstract

Supplementary Movie S1 **VSD recording of activity in a transverse slice of the IC in response to a single LL shock**. Movie corresponds to Figure 7.

Supplementary Movie S2 **VSD recording of activity in normal ACSF**. Movie corresponds to Figure 9A.

Supplementary Movie S3 **VSD recording of activity in SR95531**. Movie corresponds to Figure 9B.

Supplementary Movie S4 **VSD recording of activity in a laminar slice in response to a single LL shock**. Movie corresponds to Figure 10.

Supplementary Movie S5 **VSD recording of activity in a transverse IC slice evoked by an LL stimulus train**. Movie corresponds to Figure 13C.

Supplementary Movie S6 **VSD recording of activity in a transverse IC slice to a stimulus that evoked a circuit involving post-inhibitory rebound neurons**. Movie corresponds to Figure 14B.
